# Supplementary material for: Dissecting Mechanisms of Melanoma Resistance to BRAF and MEK Inhibitors Revealed Genetic and Non-Genetic Patient- and Drug-Specific Alterations and Remarkable Phenotypic Plasticity
Source: Cells. 2020 Jan 7;9(1):142. doi: 10.3390/cells9010142 (PMC7017165; doi:10.3390/cells9010142)

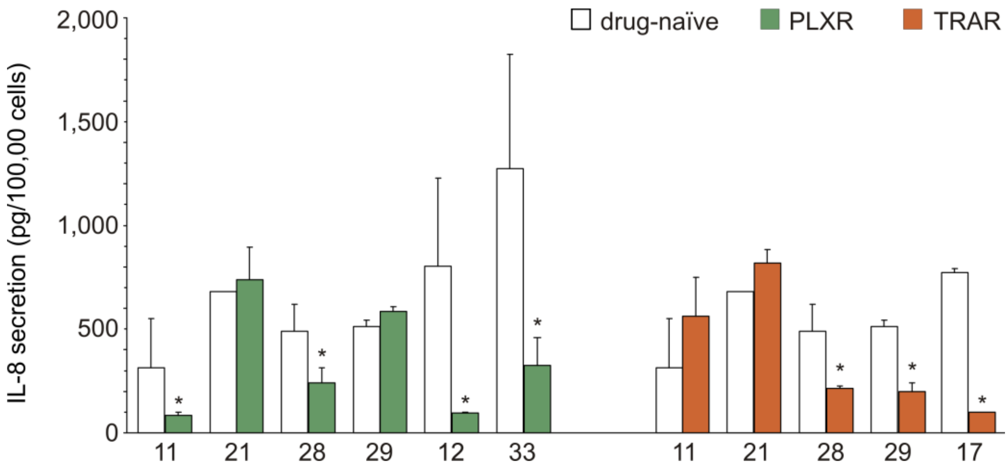

Figure S1. IL-8 levels in the culture media were assessed after 24 hours by using ELISA. Bars represent mean values of 3 biological replicates ± SD, except for 12_PLXR cells (n=2). Differences are considered significant at *p<0.05.

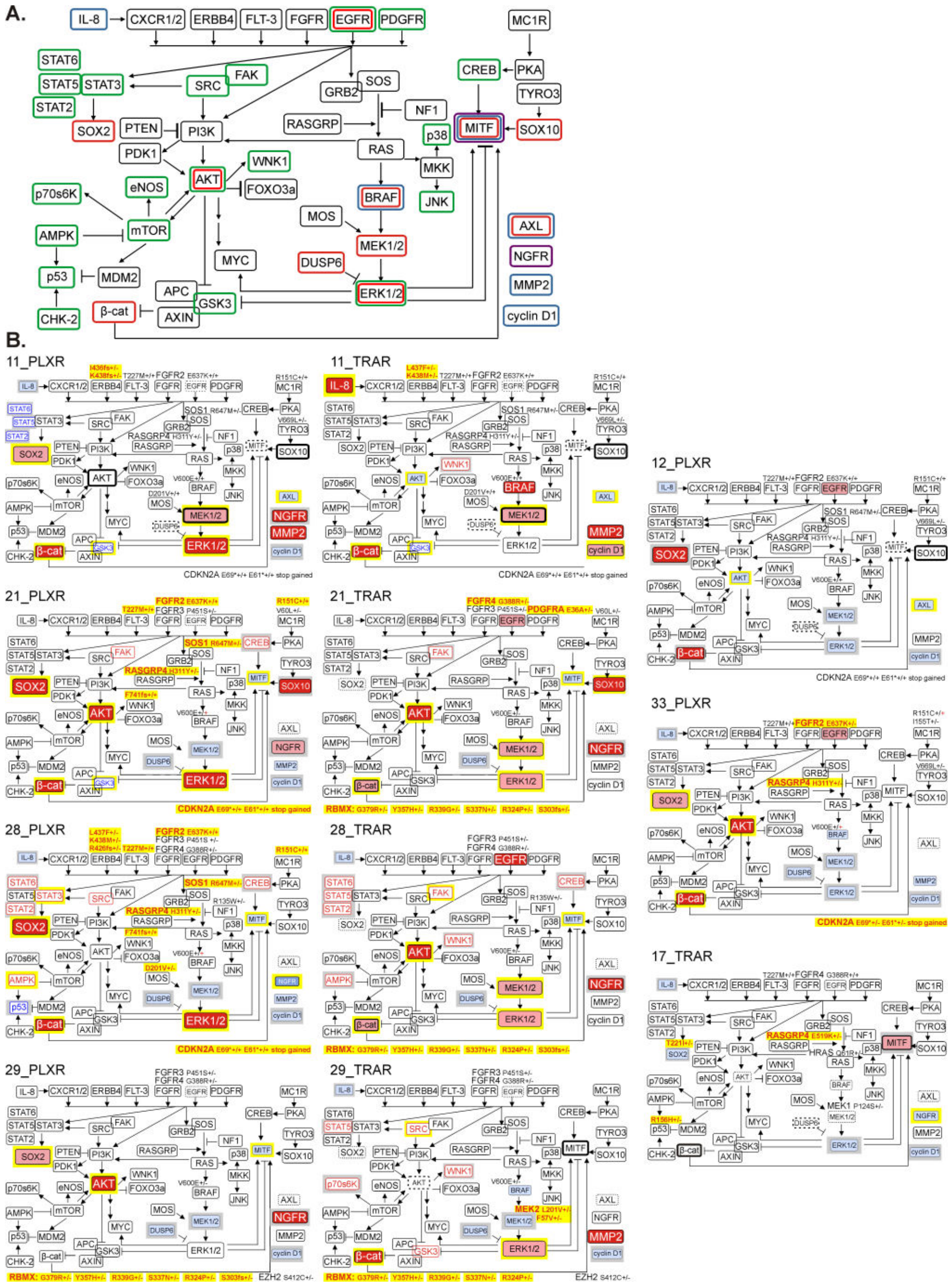

Figure S2. Diverse patterns of resistance showing patient- and drug-specific differences between drug-naïve and drug-resistant melanoma cells. (**A**) Schematic overview of signaling pathways investigated in this study with indicated method of data acquisition. Green frame, phospho-profiling; red frame, Western blotting; blue frame, qRT-PCR; violet frame, flow cytometry; black frame, not analyzed for expression or activity. In addition, all genes encoding proteins shown in the scheme were checked for the potential mutations. (**B**) Color coded overview comparing all tested cell lines in terms of differences between drug-naïve and drug-resistant cells (enlarged panels are shown in Figure 7 for selected cell lines). Red/pink, strong/weak enhancement; blue, inhibition; red/blue frame and font, enhancement/inhibition shown only in phospho-profiling; dotted frame smaller in size, weak level/activity in drug-naïve cells; bold frame, high level/activity in drug-naïve cells; mutations acquired by resistant cells are marked in red, and those already present in drug-naïve cells are marked in black. Changes, which were already induced during immediate response to drugs are in the grey background, whereas changes unique for resistant cells (including mutations) are in the yellow background.

Table S1. Mutation status of genes associated with the regulation of signaling pathways, including MAPK, PI3K/AKT and cell cycle based on the KEGG PATHWAY databases, as well as receptors and transcription factors involved in the execution of cell programs essential for melanoma progression and response to drugs. References for mutations previously implicated in resistance to MAPK inhibition are indicated in upper indices. Only non-synonymous mutations and indels are included, and shown for drug-naïve (DN), vemurafenib-resistant (PLXR) and trametinib-resistant (TRAR) cell lines. Mutations are marked as homozygous (+/+) or heterozygous (+/-). Prediction of functional effects of amino acid substitutions were assessed by using Polyphen-2 software, and were classified as benign (scores 0.000-0.449), possibly damaging (scores 0.450-0.959) and probably damaging (scores 0.960-1.000). Name of protein is given in the brackets if it differs from gene name. R, resistant cells.

<table>
<tr><th>gene</th><th></th><th>11_PLXR</th><th>21_PLXR</th><th>28_PLXR</th><th>29_PLXR</th><th>12_PLXR</th><th>33_PLXR</th></tr>
<tr><td>ABL1</td><td></td><td></td><td></td><td></td><td></td><td></td><td></td></tr>
<tr><td>ACTG1</td><td></td><td></td><td></td><td></td><td></td><td></td><td></td></tr>
<tr><td>AK1 [1]</td><td></td><td></td><td></td><td></td><td></td><td></td><td></td></tr>
<tr><td>AKT1[2]</td><td></td><td></td><td></td><td></td><td></td><td></td><td></td></tr>
<tr><td>AKT2[3]</td><td></td><td></td><td></td><td></td><td></td><td></td><td></td></tr>
<tr><td>AKT3[2]</td><td></td><td></td><td></td><td></td><td></td><td></td><td></td></tr>
<tr><td rowspan="2">ALK</td><td>DN</td><td rowspan="2">I1461V +/+
benign 0.000</td><td></td><td rowspan="2">I1461V +/+
benign 0.000</td><td rowspan="2">I1461V +/+
benign 0.000</td><td rowspan="2">I1461V +/+
benign 0.000</td><td>I1461V +/-
benign 0.000</td></tr>
<tr><td>R</td><td>I1461V +/+
benign 0.000</td><td>I1461V +/+
benign 0.000</td></tr>
<tr><td>AMER1</td><td></td><td></td><td></td><td></td><td></td><td></td><td></td></tr>
<tr><td rowspan="2">ANKRD11[1]</td><td>DN</td><td rowspan="2"></td><td rowspan="2">D2376E +/+
benign 0.010</td><td rowspan="2">D2376E +/+
benign 0.010</td><td rowspan="2">D2376E +/+
benign 0.010</td><td rowspan="2"></td><td rowspan="2">D2376E +/+
benign 0.010</td></tr>
<tr><td>R</td></tr>
<tr><td rowspan="2">APC</td><td>DN</td><td rowspan="2"></td><td rowspan="2">V1822D +/-
benign 0.000</td><td rowspan="2">V1822D +/-
benign 0.000</td><td rowspan="2">V1822D +/-
benign 0.000</td><td rowspan="2"></td><td rowspan="2">V1822D +/-
benign 0.000</td></tr>
<tr><td>R</td></tr>
<tr><td>ARAF</td><td></td><td></td><td></td><td></td><td></td><td></td><td></td></tr>
<tr><td>ARF1</td><td></td><td></td><td></td><td></td><td></td><td></td><td></td></tr>
<tr><td>ARID1A[1]</td><td></td><td></td><td></td><td></td><td></td><td></td><td></td></tr>
<tr><td rowspan="2">ARID1B[1]</td><td>DN</td><td rowspan="2"></td><td></td><td rowspan="2"></td><td rowspan="2"></td><td></td><td></td></tr>
<tr><td>R</td><td>Y1427* +/-
stop gained</td><td>Y1427* +/-
stop gained</td><td>Y1427* +/-
stop gained</td></tr>
<tr><td>ARID2[4]</td><td></td><td></td><td></td><td></td><td></td><td></td><td></td></tr>
<tr><td rowspan="4">ARID4A[1]</td><td>DN</td><td rowspan="2">N724S +/+
benign 0.003</td><td></td><td></td><td rowspan="4"></td><td rowspan="2">N724S +/+
benign 0.003</td><td></td></tr>
<tr><td>R</td><td>N724S +/+
benign 0.003</td><td>N724S +/+
benign 0.003</td><td>N724S +/+
benign 0.003</td></tr>
<tr><td>DN</td><td rowspan="2">T779A +/+
benign 0.000</td><td></td><td></td><td rowspan="2">T779A +/+
benign 0.000</td><td>T779A +/-
benign 0.000</td></tr>
<tr><td>R</td><td>T779A +/+
benign 0.000</td><td>T779A +/+
benign 0.000</td><td>T779A +/+
benign 0.000</td></tr>
<tr><td>ARID4B[1]</td><td></td><td></td><td></td><td></td><td></td><td></td><td></td></tr>
</table>

<table>
<tr><td>ARID5B[1]</td><td></td><td></td><td></td><td></td><td></td><td></td><td></td></tr>
<tr><td rowspan="4">ASXL1</td><td>DN</td><td rowspan="2">G704R +/-
probably damaging .986</td><td></td><td></td><td rowspan="2"></td><td rowspan="2">G704R +/-
probably damaging
0.986</td><td></td></tr>
<tr><td>R</td><td>G704R +/-
probably damaging
0.986</td><td>G704R +/-
probably damaging
0.986</td><td>G704R +/-
probably damaging
0.986</td></tr>
<tr><td>DN</td><td rowspan="2">L815P +/+
benign 0.000</td><td rowspan="2">L815P +/+
benign 0.000</td><td rowspan="2">L815P +/+
benign 0.000</td><td rowspan="2">L815P +/+
benign 0.000</td><td rowspan="2">L815P +/+
benign 0.000</td><td rowspan="2">L815P +/+
benign 0.000</td></tr>
<tr><td>R</td></tr>
<tr><td>ASXL2[1]</td><td></td><td></td><td></td><td></td><td></td><td></td><td></td></tr>
<tr><td rowspan="2">ATM</td><td>DN</td><td rowspan="2">N1938S +/+
benign 0.000</td><td rowspan="2">N1938S +/+
benign 0.000</td><td rowspan="2">N1938S +/+
benign 0.000</td><td rowspan="2">N1938S +/+
benign 0.000</td><td rowspan="2">N1938S +/+
benign 0.000</td><td rowspan="2">N1938S +/+
benign 0.000</td></tr>
<tr><td>R</td></tr>
<tr><td rowspan="6">ATR[1,4]</td><td>DN</td><td rowspan="2">R2425Q +/-
benign 0.000</td><td></td><td></td><td rowspan="2"></td><td rowspan="2">R2425Q +/-
benign 0.000</td><td></td></tr>
<tr><td>R</td><td>R2425Q +/-
benign 0.000</td><td>R2425Q +/-
benign 0.000</td><td>R2425Q +/-
benign 0.000</td></tr>
<tr><td>DN</td><td rowspan="2">M211T +/+
benign 0.000</td><td>M211T +/-
benign 0.000</td><td>M211T +/-
benign 0.000</td><td rowspan="2">M211T +/-
benign 0.000</td><td rowspan="2">M211T +/+
benign 0.000</td><td rowspan="2">M211T +/-
benign 0.000</td></tr>
<tr><td>R</td><td>M211T +/+
benign 0.000</td><td>M211T +/+
benign 0.000</td></tr>
<tr><td>DN</td><td rowspan="2">N1668fs +/-
frameshift variant</td><td rowspan="2"></td><td></td><td rowspan="2"></td><td rowspan="2"></td><td rowspan="2"></td></tr>
<tr><td>R</td><td>N1668fs +/-
frameshift variant</td></tr>
<tr><td>ATRX[1]</td><td></td><td></td><td></td><td></td><td></td><td></td><td></td></tr>
<tr><td rowspan="4">ATXN2[1]</td><td>DN</td><td rowspan="2">Q188 +/+
disruptive inframe deletion</td><td rowspan="2">Q188 +/+
disruptive inframe deletion</td><td rowspan="2">Q188 +/+
disruptive inframe deletion</td><td rowspan="2">Q188 +/+
disruptive inframe deletion</td><td rowspan="2">Q188 +/+
disruptive inframe deletion</td><td rowspan="2">Q188 +/+
disruptive inframe deletion</td></tr>
<tr><td>R</td></tr>
<tr><td>DN</td><td rowspan="2">L107V +/+
benign 0.000</td><td>L107V +/-
benign 0.000</td><td>L107V +/-
benign 0.000</td><td rowspan="2">L107V +/-
benign 0.000</td><td rowspan="2">L107V +/+
benign 0.000</td><td rowspan="2">L107V +/-
benign 0.000</td></tr>
<tr><td>R</td><td>L107V +/+
benign 0.000</td><td>L107V +/+
benign 0.000</td></tr>
<tr><td>AXIN1</td><td></td><td></td><td></td><td></td><td></td><td></td><td></td></tr>
<tr><td rowspan="2">AXIN2</td><td>DN</td><td rowspan="2">P50S +/+
benign 0.000</td><td rowspan="2"></td><td rowspan="2">P50S +/+
benign 0.000</td><td rowspan="2">P50S +/+
benign 0.000</td><td rowspan="2">P50S +/+
benign 0.000</td><td rowspan="2">P50S +/+
benign 0.000</td></tr>
<tr><td>R</td></tr>
<tr><td rowspan="2">AXL</td><td>DN</td><td rowspan="2">N266D +/+
benign 0.000</td><td rowspan="2">N266D +/+
benign 0.000</td><td rowspan="2">N266D +/+
benign 0.000</td><td rowspan="2">N266D +/+
benign 0.000</td><td rowspan="2">N266D +/+
benign 0.000</td><td rowspan="2">N266D +/+
benign 0.000</td></tr>
<tr><td>R</td></tr>
<tr><td>B2M[1]</td><td></td><td></td><td></td><td></td><td></td><td></td><td></td></tr>
<tr><td>BACH2</td><td></td><td></td><td></td><td></td><td></td><td></td><td></td></tr>
<tr><td>BAP1</td><td></td><td></td><td></td><td></td><td></td><td></td><td></td></tr>
<tr><td>BCL2L11 (BIM)[1]</td><td></td><td></td><td></td><td></td><td></td><td></td><td></td></tr>
<tr><td>BCL10[1]</td><td></td><td></td><td></td><td></td><td></td><td></td><td></td></tr>
<tr><td rowspan="2">BCL11B</td><td>DN</td><td rowspan="2">V690M +/-
benign 0.082</td><td></td><td></td><td rowspan="2"></td><td></td><td></td></tr>
<tr><td>R</td><td>V690M +/-</td><td>V690M +/-</td><td>V690M +/-</td><td>V690M +/-</td></tr>
</table>

			benign 0.082	benign 0.082		benign 0.082	benign 0.082
BCOR							
BCORL1[1]	DN	F111L +/+ benign 0.000	F111L +/+ benign 0.000	F111L +/+ benign 0.000	F111L +/+ benign 0.000	F111L +/+ benign 0.000	F111L +/+ benign 0.000
	R						
BLM[1]							
BMPR1A[1]	DN						P2T +/- benign 0.000
	R						
BOP1							
BRAF [2, 5, 6]	DN	V600E +/+ probably damaging 0.971	V600E +/- probably damaging 0.971	V600E +/- probably damaging 0.971	V600E +/- probably damaging 0.971	V600E +/+ probably damaging 0.971	V600E +/- probably damaging 0.971
	R		V600E +/+ probably damaging 0.971	V600E +/+ probably damaging 0.971			V600E +/+ probably damaging 0.971
BRCA1[1]	DN	S1634G +/- benign 0.002				S1634G +/- benign 0.002	S1634G +/- benign 0.002
	R		S1634G +/- benign 0.002	S1634G +/- benign 0.002			
	DN	K1183R +/- benign 0.000				K1183R +/- benign 0.000	K1183R +/- benign 0.000
	R		K1183R +/- benign 0.000	K1183R +/- benign 0.000			
	DN	E1038G +/- benign 0.012				E1038G +/- benign 0.012	E1038G +/- benign 0.012
	R		E1038G +/- benign 0.012	E1038G +/- benign 0.012			
	DN	P871L +/- benign 0.000				P871L +/- benign 0.000	P871L +/- benign 0.000
	R		P871L +/- benign 0.000	P871L +/- benign 0.000			
BRCA2	DN	V2466A +/+ benign 0.000	V2466A +/+ benign 0.000	V2466A +/+ benign 0.000	V2466A +/+ benign 0.000	V2466A +/+ benign 0.000	V2466A +/+ benign 0.000
	R						
	DN		T1915M +/- benign 0.000	T1915M +/- benign 0.000	T1915M +/- benign 0.000		T1915M +/- benign 0.000
	R				T1915M +/+ benign 0.000		
BRIP1[1]	DN	S919P +/+ benign 0.000	S919P +/- benign 0.000	S919P +/+ benign 0.000	S919P +/+ benign 0.000	S919P +/+ benign 0.000	S919P +/- benign 0.000
	R		S919P +/+ benign 0.000				S919P +/+ benign 0.000
CALR[1]							
CARD11							
CBL							
CCNA1 (cyclin A1)							
CCNA2 (cyclin A2)	DN	I163V +/+	I163V +/-	I163V +/-	I163V +/-	I163V +/+	I163V +/-

		benign 0.000	benign 0.000	benign 0.000	benign 0.000	benign 0.000	benign 0.000
	R		I163V +/+ benign 0.000				
CCNB1 (cyclin B1)							
CCNB2 (cyclin B2)							
CCND1 (cyclin D1)[7]							
CCND2 (cyclin D2)							
CCND3 (cyclin D3)	DN	S259A +/+ benign 0.000				S259A +/+ benign 0.000	S259A +/+ benign 0.000
	R		S259A +/+ benign 0.000	S259A +/+ benign 0.000			
CCNE1 (cyclin E1)							
CCNE2 (cyclin E2)							
CCNH (cyclin H)	DN		V270A +/- probably damaging 0.965	V270A +/- probably damaging 0.965	V270A +/- probably damaging 0.965		
	R						
CDC25A							
CDC25B							
CDC73 [1, 4]							
CD79B							
CDH1[1]							
CDK1							
CDK2							
CDK4							
CDK6							
CDK7							
CDK12[1]							
CDKN1A (p21)							
CDKN1B (p27)	DN	V109G +/- benign 0.047					V109G +/- benign 0.047
	R		V109G +/- benign 0.047	V109G +/- benign 0.047		V109G +/- benign 0.047	
CDKN2A (p16)[2]	DN	E69* +/+ stop gained E61* +/+ stop gained				E69* +/+ stop gained E61* +/+ stop gained	
	R		E69* +/+ stop gained E61* +/+ stop gained	E69* +/+ stop gained E61* +/+ stop gained			E69* +/- stop gained E61* +/- stop gained
CDKN2B (p15)							
CDKN2C (p18)							
CDKN2D (p19)							
CENPA[1]							
CHEK1 (Chk-1)[1]	DN	I471V +/+	I471V +/+	I471V +/+	I471V +/+	I471V +/+	I471V +/- benign 0.000

	R	benign 0.000	benign 0.000	benign 0.000	benign 0.000	benign 0.000	I471V +/+<br>benign 0.000
CHEK2 (Chk-2)							
CIC[1]							
CIITA	DN	R175G +/+<br>benign 0.000	R175G +/+<br>benign 0.000	R175G +/+<br>benign 0.000	R175G +/+<br>benign 0.000	R175G +/+<br>benign 0.000	R175G +/+<br>benign 0.000
	R						
	DN	Q901R +/+<br>benign 0.001	Q901R +/+<br>benign 0.001	Q901R +/+<br>benign 0.001	Q901R +/+<br>benign 0.001	Q901R +/+<br>benign 0.001	Q901R +/+<br>benign 0.001
	R						
	DN		G501A +/+<br>benign 0.001	G501A +/+<br>benign 0.001	G501A +/+<br>benign 0.001		
	R						
CRBN							
CREB							
CREBBP[1]	DN	V1650G +/-<br>benign 0.183					
	R						
CRLF2							
CTCF[4]							
CTLA4	DN		T17A +/-<br>benign 0.015	T17A +/-<br>benign 0.015	T17A +/-<br>benign 0.015		
	R						T17A +/-<br>benign 0.015
CTNNB1 (β-catenin)							
CTR9	DN					S477* +/- stop gained	
	R		S477* +/- stop gained				S477* +/- stop gained
CUX1[1]	DN						
	R	A448T +/-<br>benign 0.127	A448T +/-<br>benign 0.127	A448T +/-<br>benign 0.127	A448T +/-<br>benign 0.127	A448T +/-<br>benign 0.127	A448T +/-<br>benign 0.127
CXCL8 (IL-8)							
CXCR1							
CXCR2							
DAXX[1]							
DDX3X[1]							
DDX53	DN	M381I +/+<br>probably damaging<br>1.000	M381I +/-<br>probably damaging<br>1.000			M381I +/+<br>probably damaging<br>1.000	
	R		M381I +/+<br>probably damaging<br>1.000	M381I +/+<br>probably damaging<br>1.000			M381I +/+<br>probably damaging<br>1.000
	DN	R391M +/+<br>benign 0.000	R391M +/-<br>benign 0.000			R391M +/+<br>benign 0.000	
	R		R391M +/+	R391M +/+			R391M +/+

			benign 0.000	benign 0.000			benign 0.000
DEK	DN		S681P +/- benign 0.000		S681P +/- benign 0.000		S681P +/- benign 0.000
	R						
DICER1[1,4]							
DNMT3A							
DUSP1 (MKP1)							
DUSP4 (MKP2)[8]							
DUSP6 (MKP3)	DN	V114L +/- benign 0.075	V114L +/+ benign 0.075	V114L +/+ benign 0.075	V114L +/+ benign 0.075	V114L +/- benign 0.075	
	R						V114L +/- benign 0.075
	DN						S144A +/+ benign 0.000
	R						
DUSP7 (MKPX)							
DUSP10 (MKP5)							
DUSP14 (MKP6)							
DUSP16 (MKP7)	DN					V366M +/- benign 0.005	
	R	V366M +/- benign 0.005	V366M +/- benign 0.005	V366M +/- benign 0.005			V366M +/- benign 0.005
DUSP22							
E2F1							
E2F3	DN	D148N +/- possibly damaging 0.915 D389N +/- benign 0.017				D148N +/- possibly damaging 0.915 D389N +/- benign 0.017	
	R		D148N +/- possibly damaging 0.915	D389N +/- benign 0.017			
ECT2L	DN	K676fs +/- frameshift variant				K676fs +/- frameshift variant	
	R		K676fs +/- frameshift variant	K676fs +/- frameshift variant			K676fs +/- frameshift variant
EED							
EIF1AX[1]							
ELF3							
ELK1							
EGFR	DN	R521K +/- benign 0.000	R521K +/- benign 0.000	R521K +/- benign 0.000	R521K +/- benign 0.000	R521K +/- benign 0.000	
	R						R521K +/- benign 0.000
EP300[1]	DN	I997V +/+ benign 0.000				I997V +/+ benign 0.000	
	R		I997V +/+ benign 0.000	I997V +/+ benign 0.000			I997V +/+ benign 0.000
	DN	Q2223P +/- benign 0.000				Q2223P +/- benign 0.000	
	R		Q2223P +/- benign 0.000	Q2223P +/- benign 0.000			Q2223P +/- benign 0.000

	DN						
	R				N2209 +/-<br>disruptive inframe deletion		
EP400[1]	DN		A574V +/-<br>possibly damaging 0.854	A574V +/-<br>possibly damaging 0.854	A574V +/-<br>possibly damaging 0.854		
	R						
	DN		A3094T +/-<br>benign 0.001	A3094T +/-<br>benign 0.001	A3094T +/-<br>benign 0.001		
	R						
	DN		Q2742 +/-<br>disruptive inframe insertion	Q2742 +/-<br>disruptive inframe insertion	Q2742 +/-<br>disruptive inframe insertion		
	R						
EPHA3[1]	DN	W924R +/-<br>benign 0.000	W924R +/-<br>benign 0.000		W924R +/-<br>benign 0.000	W924R +/-<br>benign 0.000	W924R +/-<br>benign 0.000
	R			W924R +/-<br>benign 0.000			
EPHA7[1]							
EPHB1[1]							
ERBB2	DN		P8T +/-<br>benign 0.000	P8T +/+<br>benign 0.000	P8T +/+<br>benign 0.000		
	R						
	DN	P1170A +/-<br>possibly damaging 0.953	P1170A +/+<br>possibly damaging 0.953	P1170A +/+<br>possibly damaging 0.953	P1170A +/+<br>possibly damaging 0.953	P1170A +/-<br>possibly damaging 0.953	P1170A +/-<br>possibly damaging 0.953
	R						
ERBB3	DN						P30L +/-<br>benign 0.007
	R						
ERBB4	DN						
	R	I436fs +/-<br>frameshift variant<br>K438fs +/-<br>frameshift variant		L437F +/-<br>possibly damaging 0.928<br>K438M +/-<br>possibly damaging 0.894<br>R426 +/-<br>disruptive inframe insertion			
ERCC2	DN		D312N +/-<br>benign 0.065	D312N +/-<br>benign 0.065	D312N +/-<br>benign 0.065		D312N +/+<br>benign 0.065
	R	D312N +/-<br>benign 0.065					
	DN		K751Q +/-<br>benign 0.000	K751Q +/-<br>benign 0.000	K751Q +/-<br>benign 0.000		K751Q +/-<br>benign 0.000
	R						
ERF							
ERRFI1							
ESCO2[1]							
ETS2[3]	DN	A19T +/-<br>?				A19T +/-<br>?	
	R						

ETV6							
EZH2	DN				S412C +/- probably damaging 1.000		
	R						
FAM58A[1]	DN	A18G +/+ frameshift variant	A18G +/+ frameshift variant	A18G +/+ frameshift variant		A18G +/+ frameshift variant	A18G +/+ frameshift variant
	R						
	DN	A6G +/+ frameshift variant		A6G +/+ frameshift variant			A6G +/+ frameshift variant
	R				A6G +/+ frameshift variant		
FANCA[1]	DN	T266A +/+ benign 0.000				T266A +/+ benign 0.000	T266A +/+ benign 0.000
	R		T266A +/+ benign 0.000	T266A +/+ benign 0.000			
FANCD2[1]	DN	N405S +/- benign 0.022	N405S +/- benign 0.022	N405S +/- benign 0.022	N405S +/- benign 0.022	N405S +/- benign 0.022	
	R						N405S +/- benign 0.022
FAS[1]							
FAT1[1]	DN	K4059N +/+ benign 0.000	K4059N +/+ benign 0.000	K4059N +/+ benign 0.000	K4059N +/+ benign 0.000	K4059N +/+ benign 0.000	K4059N +/+ benign 0.000
	R						
	DN	Q2933P +/+ benign 0.000	Q2933P +/+ benign 0.000	Q2933P +/+ benign 0.000	Q2933P +/+ benign 0.000	Q2933P +/+ benign 0.000	Q2933P +/+ benign 0.000
	R						
	DN	R1064G +/+ benign 0.000	R1064G +/+ benign 0.000	R1064G +/+ benign 0.000	R1064G +/+ benign 0.000	R1064G +/+ benign 0.000	R1064G +/- benign 0.000
	R						R1064G +/+ benign 0.000
	DN	V862L +/+ benign 0.000	V862L +/+ benign 0.000	V862L +/+ benign 0.000	V862L +/+ benign 0.000	V862L +/+ benign 0.000	V862L +/- benign 0.000
	R						V862L +/+ benign 0.000
	DN	F614L +/+ benign 0.000	F614L +/+ benign 0.000	F614L +/+ benign 0.000	F614L +/+ benign 0.000	F614L +/+ benign 0.000	F614L +/- benign 0.000
	R						F614L +/+ benign 0.000
	DN	S404R +/+ benign 0.032	S404R +/+ benign 0.032	S404R +/+ benign 0.032	S404R +/+ benign 0.032	S404R +/+ benign 0.032	S404R +/- benign 0.032
	R						S404R +/+ benign 0.032
	DN	V482I +/+ benign 0.009	V482I +/+ benign 0.009	V482I +/+ benign 0.009	V482I +/+ benign 0.009	V482I +/+ benign 0.009	V482I +/- benign 0.009
	R						V482I +/+ benign 0.009
	DN	H1273R +/+				H1273R +/+	H1273R +/-

		benign 0.000				benign 0.000	benign 0.000
	R		H1273R +/+<br>benign 0.000	H1273R +/+<br>benign 0.000			H1273R +/+<br>benign 0.000
FBXO11							
FBXW7[1]	DN	V418M +/+<br>possibly damaging 0.801				V418M +/-<br>possibly damaging 0.801	
	R		V418M +/-<br>possibly damaging 0.801	V418M +/-<br>possibly damaging 0.801			V418M +/-<br>possibly damaging 0.801
FGFR1							
FGFR2	DN	E637K +/+<br>probably damaging<br>0.970				E637K +/+<br>probably damaging<br>0.970	
	R		E637K +/+<br>probably damaging<br>0.970	E637K +/+<br>probably damaging<br>0.970			E637K +/-<br>probably damaging<br>0.970
FGFR3	DN		P451S +/-<br>possibly damaging 0.902	P451S +/-<br>possibly damaging 0.902	P451S +/-<br>possibly damaging 0.902		
	R						
FGFR4	DN	V10I +/-<br>benign 0.000				V10I +/-<br>benign 0.000	
	R		V10I +/-<br>benign 0.000	V10I +/-<br>benign 0.000			V10I +/-<br>benign 0.000
	DN	P136L +/-<br>benign 0.000	P136L +/-<br>benign 0.000	P136L +/-<br>benign 0.000<br>G388R +/-<br>probably damaging<br>0.998	P136L +/-<br>benign 0.000<br>G388R +/-<br>probably damaging<br>0.998	P136L +/-<br>benign 0.000	P136L +/-<br>benign 0.000
	R						
FH							
FLT3	DN	T227M +/+<br>probably damaging<br>0.999				T227M +/+<br>probably damaging<br>0.999	T227M +/-<br>probably damaging<br>0.999
	R		T227M +/+<br>probably damaging<br>0.999	T227M +/+<br>probably damaging<br>0.999			
FOXA1							
FOXM1	DN						
	R				S681P +/-<br>benign 0.000		
FOXO3A							
FOXP1[1]							
GADD45A							
GADD45B							
GADD45G							
GNA11							
GNAS[1, 4]	DN					P459R +/-<br>benign 0.005	
	R		P459R +/-<br>benign 0.005	P459R +/-<br>benign 0.005			

	DN	A436D +/- benign 0.001				A436D +/- benign 0.001	
	R		A436D +/- benign 0.001	A436D +/- benign 0.001			A436D +/- benign 0.001
GNB1[1]							
GRB2							
GRIN2A[1, 4]							
GSK3A							
GSK3B							
HDAC4							
HIST1H2BC[1]							
HIST1H3H[1]							
HLA-A[1]							
HLA-B							
HNF1A	DN	S574G +/+ benign 0.000	S574G +/+ benign 0.000	S574G +/+ benign 0.000	S574G +/+ benign 0.000	S574G +/+ benign 0.000	S574G +/+ benign 0.000
	R						
	DN		I27L +/- benign 0.002	I27L +/- benign 0.002	I27L +/- benign 0.002		
	R						
	DN		S487N +/- benign 0.000	S487N +/- benign 0.000	S487N +/- benign 0.000		
	R						
HOXD8[9]							
HRAS							
IDH1[4]							
IDH2[4]							
IGF1R							
IGFBP7	DN						
	R	L11F +/- benign 0.005	L11F +/+ benign 0.005	L11F +/+ benign 0.005		L11F +/- benign 0.005	L11F +/+ benign 0.005
INPP4B							
INPPL1	DN	L632I +/- probably damaging 0.990				L632I +/- probably damaging 0.990	
	R		L632I +/- probably damaging 0.990	L632I +/- probably damaging 0.990			L632I +/- probably damaging 0.990
	DN	A1083G +/- benign 0.000				A1083G +/- benign 0.000	
	R		A1083G +/- benign 0.000	A1083G +/- benign 0.000			A1083G +/- benign 0.000
IRF8 [1]							
JAK1 [1]							
JAK2							
JARID2 [1]							

KDM5C							
KDM6A	DN	T778K +/+<br>benign 0.000				T778K +/+<br>benign 0.000	
	R						
KDR (VEGFR2)[4]	DN		Q472H +/-<br>benign 0.003	Q472H +/-<br>benign 0.003	Q472H +/-<br>benign 0.003		Q472H +/-<br>benign 0.003
	R						
	DN		V297I +/-<br>probably damaging<br>1.000	V297I +/-<br>probably damaging<br>1.000	V297I +/-<br>probably damaging<br>1.000		
	R						
KEAP1							
KIT							
KMT2A[1]	DN		A30G +/+<br>possibly damaging<br>0.953	A30G +/+<br>possibly damaging<br>0.953	A30G +/+<br>possibly damaging<br>0.953		
	R						
KMT2B[1]	DN	G296S +/-<br>probably damaging<br>1.000				G296S +/-<br>probably damaging<br>1.000	
	R		G296S +/+<br>probably damaging<br>1.000	G296S +/-<br>probably damaging<br>1.000			G296S +/+<br>probably damaging<br>1.000
	DN	D2364G +/-<br>benign 0.000	D2364G +/-<br>benign 0.000	D2364G +/-<br>benign 0.000	D2364G +/-<br>benign 0.000	D2364G +/-<br>benign 0.000	D2364G +/+<br>benign 0.000
	R						
	DN	R1021fs +/+<br>frameshift variant	R1021fs +/+<br>frameshift variant	R1021fs +/+<br>frameshift variant	R1021fs +/+<br>frameshift variant	R1021fs +/+<br>frameshift variant	R1021fs +/+<br>frameshift variant
	R						
	DN						P1829L +/-<br>benign 0.006
	R						
KMT2C[1,4]	DN						
	R	C988F +/-<br>probably damaging<br>0.999	C988F +/-<br>probably damaging<br>0.999	C988F +/-<br>probably damaging<br>0.999	C988F +/-<br>probably damaging<br>0.999	C988F +/-<br>probably damaging<br>0.999	C988F +/-<br>probably damaging<br>0.999
	DN						
	R	T316S +/-<br>possibly damaging 0.684	T316S +/-<br>possibly damaging 0.684	T316S +/-<br>possibly damaging 0.684	T316S +/-<br>possibly damaging 0.684	T316S +/-<br>possibly damaging 0.684	T316S +/-<br>possibly damaging 0.684
	DN						
	R	L291F +/-<br>probably damaging<br>1.000	L291F +/-<br>probably damaging<br>1.000	L291F +/-<br>probably damaging<br>1.000	L291F +/-<br>probably damaging<br>1.000	L291F +/-<br>probably damaging<br>1.000	L291F +/-<br>probably damaging<br>1.000
	DN						
	R	Y816X +/-<br>frameshift variant	Y816X +/-<br>frameshift variant	Y816X +/-<br>frameshift variant	Y816X +/-<br>frameshift variant	Y816X +/-<br>frameshift variant	Y816X +/-<br>frameshift variant
	DN						
	R			G838S +/-<br>probably damaging			

				1.000			
	DN						
	R				S772L +/-<br>possibly damaging 0.546		
	DN						
	R				R284Q +/-<br>probably damaging<br>0.984		
	DN						
	R				N729D +/-<br>benign 0.094		
KMT2D[1, 4]	DN	V4305I +/+<br>benign 0.039				V4305I +/-<br>benign 0.039	
	R						
	DN		P813L +/-<br>benign 0.000	P813L +/-<br>benign 0.000	P813L +/-<br>benign 0.000		
	R						
KNSTRN	DN	N279Y +/-<br>probably damaging<br>0.965				N279Y +/-<br>probably damaging<br>0.965	
	R		N279Y +/-<br>probably damaging<br>0.965	N279Y +/-<br>probably damaging<br>0.965			N279Y +/-<br>probably damaging<br>0.965
KRAS[2, 3, 10]							
LAMTOR1 (MP1)							
LATS1[1]							
MAP2K1 (MEK1)[9, 11-13]							
MAP2K2 (MEK2)[3, 9, 14]							
MAP2K4[1]							
MAPK3 (ERK1)							
MAPK1 (ERK2)							
MAPK8 (JNK1)							
MAPK9 (JNK2)							
MAPK10 (JNK3)							
MAPK14 (p38)							
MC1R	DN	R151C +/+<br>probably damaging<br>1.000				R151C +/+<br>probably damaging<br>1.000	R151C +/-<br>probably damaging<br>1.000
	R		R151C +/+<br>probably damaging<br>1.000	R151C +/+<br>probably damaging<br>1.000			R151C +/+<br>probably damaging<br>1.000

	DN		V60L +/-<br>probably damaging 0.988				I155T +/-<br>probably damaging 0.986
	R						
MDM2							
MDM4							
MED12[1]	DN	H2116 +/+<br>disruptive inframe insertion				H2116 +/-<br>disruptive inframe insertion	
	R		H2116 +/+<br>disruptive inframe insertion	H2116 +/+<br>disruptive inframe insertion		H2116 +/+<br>disruptive inframe insertion	H2116 +/+<br>disruptive inframe insertion
MEN1[4]	DN	T546A +/+<br>benign 0.000	T546A +/+<br>benign 0.000	T546A +/+<br>benign 0.000	T546A +/+<br>benign 0.000	T546A +/+<br>benign 0.000	T546A +/+<br>benign 0.000
	R						
MET							
MGA[1]	DN	T716S +/-<br>benign 0.000	T716S +/+<br>benign 0.000	T716S +/+<br>benign 0.000	T716S +/+<br>benign 0.000	T716S +/-<br>benign 0.000	T716S +/+<br>benign 0.000
	R						
	DN		P1523A +/+<br>possibly damaging 0.657	P1523A +/+<br>possibly damaging 0.657	P1523A +/+<br>possibly damaging 0.657		
	R						
MITF[9]							
MKI67 (Ki67)	DN	I2101T +/+<br>benign 0.008	I2101T +/-<br>benign 0.008	I2101T +/-<br>benign 0.008	I2101T +/-<br>benign 0.008	I2101T +/+<br>benign 0.008	
	R		I2101T +/+<br>benign 0.008	I2101T +/+<br>benign 0.008			
	DN	N104S +/+<br>probably damaging 0.982	N104S +/+<br>probably damaging 0.982<br>T2868S +/-<br>probably damaging 0.999<br>R2786Q +/-<br>probably damaging 0.991<br>E1403V +/-<br>probably damaging 0.997<br><br>K3217E +/-<br>benign 0.000<br>T3150S +/-<br>benign 0.000<br>N2363S +/-	N104S +/+<br>probably damaging 0.982<br>T2868S +/-<br>probably damaging 0.999<br>R2786Q +/-<br>probably damaging 0.991<br>E1403V +/-<br>probably damaging 0.997<br><br>K3217E +/-<br>benign 0.000<br>T3150S +/-<br>benign 0.000<br>N2363S +/-	N104S +/+<br>probably damaging 0.982<br>T2868S +/-<br>probably damaging 0.999<br>R2786Q +/-<br>probably damaging 0.991<br>E1403V +/-<br>probably damaging 0.997<br><br>K3217E +/-<br>benign 0.000<br>T3150S +/-<br>benign 0.000<br>N2363S +/-	N104S +/+<br>probably damaging 0.982	R2786Q +/-<br>probably damaging 0.991<br><br>T3150S +/-<br>benign 0.000<br>N2363S +/-<br>benign 0.000
	R						

			benign 0.000 G1042S +/- benign 0.401	benign 0.000 G1042S +/- benign 0.401	benign 0.000 G1042S +/- benign 0.401		
MLH1[1]	DN		I219V +/- benign 0.018				I219V +/- benign 0.018
	R						
MMP2							
MOS	DN	D201V +/+ probably damaging 1.000					
	R			D201V +/- probably damaging 1.000			
MPL[1]							
MRE11A[1]							
MSH2							
MSH3[1]	DN	Q949R +/+ benign 0.000	Q949R +/+ benign 0.000	Q949R +/+ benign 0.000	Q949R +/+ benign 0.000	Q949R +/+ benign 0.000	
	R						Q949R +/+ benign 0.000
	DN	A1045T +/- benign 0.075	A1045T +/- benign 0.075	A1045T +/- benign 0.075	A1045T +/- benign 0.075	A1045T +/- benign 0.075	
	R						A1045T +/- benign 0.075
	DN	A61-P63dup +/- inframe insertion	A61-P63dup +/- inframe insertion	A61-P63dup +/- inframe insertion	A61-P63dup +/- inframe insertion	A61-P63dup +/- inframe insertion	A61-P63dup +/- inframe insertion
	R						
	DN						P72S +/- benign 0.028
	R						
MSH6[1]	DN		G39E +/- benign 0.000	G39E +/- benign 0.000	G39E +/- benign 0.000		
	R						
MTOR	DN	R2152C +/- benign 0.050				R2152C +/- benign 0.050	
	R		R2152C +/- benign 0.050	R2152C +/- benign 0.050			
MUTYH[1]	DN	Q338H +/- benign 0.343	Q338H +/+ benign 0.343	Q338H +/+ benign 0.343	Q338H +/+ benign 0.343		Q338H +/- benign 0.343
	R					Q338H +/- benign 0.343	
MYC							
MYD88[1]							
MYT1	DN		T782S +/- benign 0.035	T782S +/- benign 0.035	T782S +/- benign 0.035		
	R						
NBN [1]	DN	E185Q +/- benign 0.001				E185Q +/- benign 0.001	
	R		E185Q +/- benign 0.001	E185Q +/- benign 0.001			E185Q +/- benign 0.001
NCOR1[1]							

NF1[15]	DN			R135W +/-<br>probably damaging<br>1.000			
	R						
NF2[1]							
NFKBIA							
NGFR							
NOS3 (eNOS)	DN	D298E +/+<br>benign 0.000	D298E +/-<br>benign 0.000	D298E +/-<br>benign 0.000	D298E +/-<br>benign 0.000	D298E +/+<br>benign 0.000	D298E +/-<br>benign 0.000
	R		D298E +/+<br>benign 0.000	D298E +/+<br>benign 0.000			D298E +/+<br>benign 0.000
NOTCH1[1]							
NOTCH2[1]	DN	F1209V +/-<br>possibly damaging 0.939				F1209V +/-<br>possibly damaging 0.939	
	R		F1209V +/-<br>possibly damaging 0.939	F1209V +/-<br>possibly damaging 0.939			F1209V +/-<br>possibly damaging 0.939
	DN						N2002S +/-<br>probably damaging .999
	R						
	DN						
	R	N46S +/-<br>benign 0.010	N46S +/-<br>benign 0.010	N46S +/-<br>benign 0.010	N46S +/-<br>benign 0.010	N46S +/-<br>benign 0.010	N46S +/-<br>benign 0.010
	DN						
	R	E38K +/-<br>benign 0.044	E38K +/-<br>benign 0.044	E38K +/-<br>benign 0.044	E38K +/-<br>benign 0.044	E38K +/-<br>benign 0.044	E38K +/-<br>benign 0.044
	DN						
	R	C19W +/-<br>benign 0.001	C19W +/-<br>benign 0.001	C19W +/-<br>benign 0.001	C19W +/-<br>benign 0.001	C19W +/-<br>benign 0.001	C19W +/-<br>benign 0.001
	DN						
	R				A21T +/-<br>benign 0.000		
NOTCH3	DN	C1826F +/-<br>possibly damaging 0.829				C1826F +/-<br>possibly damaging 0.829	
	R		C1826F +/-<br>possibly damaging 0.829	C1826F +/-<br>possibly damaging 0.829			C1826F +/-<br>possibly damaging 0.829
	DN	A2223V +/+<br>benign 0.001	A2223V +/+<br>benign 0.001		A2223V +/+<br>benign 0.001	A2223V +/+<br>benign 0.001	A2223V +/+<br>benign 0.001
	R						
NOTCH4[4]							
NRAS[2, 12]							
NSD1[1]							
NTRK1							
NTRK2							
NTRK3							
PAK5[1]							

PALB2[1]							
PARK2[1]							
PARP1[1]							
PAX5[1]	DN	G266E +/+<br>benign 0.000				G266E +/+<br>benign 0.000	
	R		G266E +/+<br>benign 0.000	G266E +/+<br>benign 0.000			G266E +/+<br>benign 0.000
	DN		T293I +/+<br>benign 0.000	T293I +/+<br>benign 0.000	T293I +/+<br>benign 0.000		T293I +/+<br>benign 0.000
	R						
PBRM1[1, 4]							
PDGFRA							
PDGFRB							
PDPK1 (PDK1)							
PHLPP1[2]							
PIGA[1]							
PIK3CA[2]							
PIK3CB	DN						R981L +/-<br>possibly damaging 0.601
	R						
PIK3CG[2]							
PIK3R1							
PIK3R2[2]	DN	S313P +/+<br>benign 0.000<br>S234R +/+<br>benign 0.000	S313P +/+<br>benign 0.000<br>S234R +/+<br>benign 0.000	S313P +/+<br>benign 0.000<br>S234R +/+<br>benign 0.000	S313P +/+<br>benign 0.000<br>S234R +/+<br>benign 0.000	S313P +/+<br>benign 0.000<br>S234R +/+<br>benign 0.000	S313P +/+<br>benign 0.000<br>S234R +/+<br>benign 0.000
	R						
PIK3R3	DN						
	R	N283K +/+<br>benign 0.000	N283K +/+<br>benign 0.000<br>M295X +/-<br>frameshift variant	N283K +/+<br>benign 0.000	N283K +/-<br>benign 0.000	N283K +/+<br>benign 0.000<br>M295X +/-<br>frameshift variant	N283K +/+<br>benign 0.000
PMS1[1]							
PMS2[1]	DN	K541E +/+<br>benign 0.000	K541E +/-<br>benign 0.000	K541E +/-<br>benign 0.000	K541E +/-<br>benign 0.000	K541E +/+<br>benign 0.000	K541E +/+<br>benign 0.000
	R						
	DN	P470S +/-<br>benign 0.018				P470S +/-<br>benign 0.018	P470S +/-<br>benign 0.018
	R		P470S +/-<br>benign 0.018	P470S +/-<br>benign 0.018			
POT1							
PPP2R1A	DN						
	R		E332D +/-<br>benign 0.000			E332D +/-<br>benign 0.000	E332D +/-<br>benign 0.000
PPP6C [1]							
PRDM1[1]	DN	G74S +/-				G74S +/-	

	R	benign 0.016	G74S +/-<br>benign 0.016	G74S +/-<br>benign 0.016		benign 0.016	G74S +/-<br>benign 0.016
PRKAA1 (AMPK1)							
PRKAA2 (AMPK2)							
PRKACA (PKA)							
PRKACB (PKA)							
PTCH1	DN		P1315L +/-<br>possibly damaging 0.944	P1315L +/-<br>possibly damaging 0.944	P1315L +/-<br>possibly damaging 0.944		
	R						
PTEN[2]							
PTK2 (FAK)							
PTPN1							
PTPN11							
PTPRD[1, 4]	DN	G272R +/-<br>probably damaging 0.977				G272R +/-<br>probably damaging 0.977	
	R		G272R +/-<br>probably damaging 0.977	G272R +/-<br>probably damaging 0.977			G272R +/-<br>probably damaging 0.977
	DN		T781A +/-<br>benign 0.007	T781A +/-<br>benign 0.007	T781A +/-<br>benign 0.007		
	R						
PTPRS	DN	C1457R +/+<br>benign 0.000	C1457R +/+<br>benign 0.000	C1457R +/+<br>benign 0.000	C1457R +/+<br>benign 0.000	C1457R +/+<br>benign 0.000	C1457R +/+<br>benign 0.000
	R						
PTPRT[4]	DN	A29P +/-<br>benign 0.014	A29P +/+<br>benign 0.014	A29P +/+<br>benign 0.014	A29P +/+<br>benign 0.014		
	R						
RAC1[9, 16]							
RAD50[1]							
RAD51							
RAF1 (CRAF)							
RASA2							
RASGRF1							
RASGRF2	DN	S753P +/+<br>benign 0.002				S753P +/-<br>benign 0.002	
	R		S753P +/-<br>benign 0.002	S753P +/-<br>benign 0.002			S753P +/-<br>benign 0.002
RASGRP1							
RASGRP2							
RASGRP3	DN	T393A +/-<br>benign 0.131	T393A +/-<br>benign 0.131				
	R						T393A +/-<br>benign 0.131
RASGRP4	DN	H311Y +/-				H311Y +/-	

<table>
<tr><td rowspan="3"></td><td>R</td><td>probably damaging
0.993</td><td>H311Y +/-
probably damaging
0.993</td><td>H311Y +/-
probably damaging
0.9</td><td></td><td>probably damaging
0.993</td><td>H311Y +/-
probably damaging
0.993</td></tr>
<tr><td>DN</td><td rowspan="2">G165R +/-
benign 0.001
I18T +/-
benign 0.000</td><td rowspan="2"></td><td></td><td rowspan="2"></td><td rowspan="2"></td><td rowspan="2">I18T +/-
benign 0.000</td></tr>
<tr><td>R</td><td>G165R +/-
benign 0.001
I18T +/-
benign 0.000</td></tr>
<tr><td>*RASSF2*</td><td></td><td></td><td></td><td></td><td></td><td></td><td></td></tr>
<tr><td>*RB1*</td><td></td><td></td><td></td><td></td><td></td><td></td><td></td></tr>
<tr><td>*RBM10*[17]</td><td></td><td></td><td></td><td></td><td></td><td></td><td></td></tr>
<tr><td rowspan="2">*RBMX* [17]</td><td>DN</td><td></td><td></td><td></td><td></td><td></td><td></td></tr>
<tr><td>R</td><td></td><td></td><td></td><td>G379R +/-
probably damaging
1.000
Y357H +/-
probably damaging
0.995
R339G +/-
possibly damaging
0.995
S337N +/-
probably damaging
0.981
R324P +/-
probably damaging
0.996
S303fs +/-
frameshift variant
P301L +/+
benign 0.269</td><td></td><td></td></tr>
<tr><td>*RECQL*[1]</td><td></td><td></td><td></td><td></td><td></td><td></td><td></td></tr>
<tr><td rowspan="2">*RET*</td><td>DN</td><td rowspan="2">G691S +/+
benign 0.062</td><td></td><td rowspan="2"></td><td rowspan="2"></td><td rowspan="2">G691S +/+
benign 0.062</td><td></td></tr>
<tr><td>R</td><td>G691S +/+
benign 0.062</td><td>G691S +/-
benign 0.062</td></tr>
<tr><td>*RHEB*</td><td></td><td></td><td></td><td></td><td></td><td></td><td></td></tr>
<tr><td rowspan="2">*RICTOR*</td><td>DN</td><td rowspan="2"></td><td rowspan="2">S837F +/+
benign 0.002</td><td rowspan="2">S837F +/+
benign 0.002</td><td rowspan="2">S837F +/+
benign 0.002</td><td rowspan="2"></td><td rowspan="2">S837F +/+
benign 0.002
R907C +/-
possibly damaging 0.915</td></tr>
<tr><td>R</td></tr>
<tr><td>*RIT1*</td><td></td><td></td><td></td><td></td><td></td><td></td><td></td></tr>
<tr><td>*RNF43*[4]</td><td>DN</td><td>P231L +/+</td><td></td><td></td><td></td><td>P231L +/+</td><td></td></tr>
</table>

	R	benign 0.056	P231L +/+<br>benign 0.056	P231L +/+<br>benign 0.056		benign 0.056	P231L +/+<br>benign 0.056
	DN		I47V +/-<br>benign 0.002	I47V +/+<br>benign 0.002	I47V +/+<br>benign 0.002		
	R						
	DN		L418M +/-<br>probably damaging<br>0.969	L418M +/+<br>probably damaging<br>0.969	L418M +/+<br>probably damaging<br>0.969		
	R						
	DN		R343H +/-<br>benign 0.002	R343H +/-<br>benign 0.002	R343H +/-<br>benign 0.002		
	R				R343H +/+<br>benign 0.002		R343H +/-<br>benign 0.002
	DN						P686R +/-<br>benign 0.001
	R						
ROS1	DN	D2213N +/-<br>benign 0.009	D2213N +/+<br>benign 0.009	D2213N +/+<br>benign 0.009	D2213N +/+<br>benign 0.009	D2213N +/-<br>benign 0.009	
	R						D2213N +/-<br>benign 0.009
	DN	S2229C +/-<br>benign 0.000<br>K2228Q +/-<br>benign 0.000	S2229C +/+<br>benign 0.000<br>K2228Q +/+<br>benign 0.000	S2229C +/+<br>benign 0.000<br>K2228Q +/+<br>benign 0.000	S2229C +/+<br>benign 0.000<br>K2228Q +/+<br>benign 0.000	S2229C +/-<br>benign 0.000<br>K2228Q +/-<br>benign 0.000	
	R						
RPS6KA1 (RSK1)	DN	K344T +/-<br>benign 0.088				K344T +/-<br>benign 0.088	
	R		K344T +/-<br>benign 0.088	K344T +/-<br>benign 0.088			K344T +/-<br>benign 0.088
RPS6KA2 (RSK3)	DN	T34A +/+<br>benign 0.000<br>E32G +/+<br>benign 0.000	T34A +/+<br>benign 0.000<br>E32G +/+<br>benign 0.000	T34A +/+<br>benign 0.000<br>E32G +/+<br>benign 0.000	T34A +/+<br>benign 0.000<br>E32G +/+<br>benign 0.000	T34A +/+<br>benign 0.000<br>E32G +/+<br>benign 0.000	T34A +/+<br>benign 0.000<br>E32G +/+<br>benign 0.000<br>I10S +/-<br>benign 0.000
	R						
RPS6KA3 (RSK2)	DN						I38S +/-<br>benign 0.000
	R						
RPS6KA4 (MSK2)	DN	S758A +/-<br>benign 0.000	S758A +/-<br>benign 0.000	S758A +/-<br>benign 0.000	S758A +/-<br>benign 0.000	S758A +/-<br>benign 0.000	S758A +/-<br>benign 0.000
	R	S758A +/+<br>benign 0.000					
RPS6KA5 (MSK1)							
RPS6KB1 (p70S6K)							
RPTOR							
RTEL1	DN	Q1042H +/-<br>benign 0.000	Q1042H +/+<br>benign 0.000	Q1042H +/+<br>benign 0.000	Q1042H +/+<br>benign 0.000		Q1042H +/+<br>benign 0.000
	R					Q1042H +/+<br>benign 0.000	

SAMD4B [3]							
SDHA[1]							
SDHB[4]							
SDHC[1]							
SDHD							
SETD2[1, 4]	DN		P1962L +/-<br>benign 0.001				P1962L +/-<br>benign 0.001
	R				P1962L +/-<br>benign 0.001		
SF3B1[4]							
SH2D1A							
SHQ1	DN	S489N +/+<br>benign 0.000	S489N +/-<br>benign 0.000	S489N +/-<br>benign 0.000	S489N +/-<br>benign 0.000	S489N +/+<br>benign 0.000	
	R		S489N +/+<br>benign 0.000	S489N +/+<br>benign 0.000			S489N +/+<br>benign 0.000
	DN	F72C +/-<br>probably damaging<br>0.971				F72C +/-<br>probably damaging<br>0.971	
	R		F72C +/-<br>probably damaging<br>0.971	F72C +/-<br>probably damaging<br>0.971			F72C +/-<br>probably damaging<br>0.971
SLX4							
SMAD3							
SMAD4[1]							
SMARCA4 (BRG1)[1]							
SOS1	DN	R647M +/-<br>probably damaging<br>0.998				R647M +/-<br>probably damaging<br>0.998	
	R		R647M +/-<br>probably damaging<br>0.998	R647M +/-<br>probably damaging<br>0.998			
	DN						
	R				S1318C +/-<br>benign 0.145		
SOS2	DN						A208T +/-<br>possibly damaging 0.937
	R						
SOX2							
SOX9							
SOX10							
SPEN[1]	DN	A970V +/-<br>benign 0.006				A970V +/-<br>benign 0.006	
	R						
	DN	L1091P +/+<br>benign 0.000				L1091P +/+<br>benign 0.000	L1091P +/-<br>benign 0.000
	R						

	DN	N2360D +/+<br>benign 0.000				N2360D +/+<br>benign 0.000	N2360D +/-<br>benign 0.000
	R						
	DN						D2007E +/-<br>benign 0.001
	R						
	DN						
	R	L2650 +/-<br>disruptive inframe deletion	L2650 +/-<br>disruptive inframe deletion	L2650 +/-<br>disruptive inframe deletion		L2650 +/-<br>disruptive inframe deletion	L2650 +/-<br>disruptive inframe deletion
SPOP[1]							
SPRED1							
SRC							
STAG2[1]							
STAT3							
STAT5A							
STAT5B							
STK11							
STK19							
SUFU							
SYK							
TBX2							
TCF3[1]							
TCF7L2[1]							
TERT							
TET1	DN	V128F +/+<br>possibly damaging 0.845				V128F +/+<br>possibly damaging 0.845	
	R		V128F +/+<br>possibly damaging 0.845	V128F +/+<br>possibly damaging 0.845			V128F +/+<br>possibly damaging 0.845
	DN		D162G +/-<br>benign 0.295	D162G +/-<br>benign 0.295	D162G +/-<br>benign 0.295		D162G +/-<br>benign 0.295
	R						
	DN	I1123M +/+<br>benign 0.070	I1123M +/+<br>benign 0.070	I1123M +/+<br>benign 0.070	I1123M +/+<br>benign 0.070	I1123M +/+<br>benign 0.070	I1123M +/-<br>benign 0.070
	R						
	DN						S193T +/-<br>benign 0.107
	R						
TET2[4]	DN	I1762V +/+<br>benign 0.001	I1762V +/-<br>benign 0.001	I1762V +/-<br>benign 0.001	I1762V +/-<br>benign 0.001	I1762V +/+<br>benign 0.001	I1762V +/-<br>benign 0.001
	R		I1762V +/+<br>benign 0.001	I1762V +/+<br>benign 0.001			I1762V +/+<br>benign 0.001
TGFBR2							
TMEM127[1]							
TOP1	DN	H81Y +/-				H81Y +/-	

	R	benign 0.273	H81Y +/-<br>benign 0.273	H81Y +/-<br>benign 0.273		benign 0.273	H81Y +/-<br>benign 0.273
TP53	DN	P72R +/-<br>benign 0.083	P72R +/-<br>benign 0.083	P72R +/+<br>benign 0.083	P72R +/+<br>benign 0.083		P72R +/+<br>benign 0.083
	R						
TP53BP1[1]	DN	K1141Q +/-<br>benign 0.000				K1141Q +/-<br>benign 0.000	
	R		K1141Q +/-<br>benign 0.000	K1141Q +/-<br>benign 0.000			K1141Q +/-<br>benign 0.000
	DN	G417S +/-<br>benign 0.000				G417S +/-<br>benign 0.000	
	R		G417S +/-<br>benign 0.000	G417S +/-<br>benign 0.000			G417S +/-<br>benign 0.000
	DN	D358E +/-<br>benign 0.000				D358E +/-<br>benign 0.000	
	R		D358E +/-<br>benign 0.000	D358E +/-<br>benign 0.000			D358E +/-<br>benign 0.000
TP63[4]							
TSC1	DN		M322T +/-<br>benign 0.000	M322T +/-<br>benign 0.000	M322T +/-<br>benign 0.000		
	R						
TSC2							
TYRO3	DN	I346N +/-<br>benign 0.408				I346N +/-<br>benign 0.408	I346N +/-<br>benign 0.408
	R		I346N +/-<br>benign 0.408	I346N +/-<br>benign 0.408			
	DN	V669L +/-<br>probably damaging<br>1.000				V669L +/-<br>probably damaging<br>1.000	V669L +/-<br>probably damaging<br>1.000
	R						
VHL							
WEE1	DN						G210C +/-<br>possibly damaging 0.903
	R						
WNK1	DN						
	R		F741fs +/+<br>frameshift variant	F741fs +/+<br>frameshift variant			
	DN	T1316P +/-<br>benign 0.000<br>C1766S +/+<br>benign 0.000	T1316P +/+<br>benign 0.000<br>C1766S +/+<br>benign 0.000<br>M2068I +/-<br>benign 0.000	T1316P +/+<br>benign 0.000<br>C1766S +/+<br>benign 0.000<br>M2068I +/-<br>benign 0.000	T1316P +/+<br>benign 0.000<br>C1766S +/+<br>benign 0.000<br>M2068I +/-<br>benign 0.000	T1316P +/-<br>benign 0.000<br>C1766S +/+<br>benign 0.000	T1316P +/-<br>benign 0.000<br>C1766S +/+<br>benign 0.000<br>M2068I +/-<br>benign 0.000
	R						
WT1							
XRCC2[1]							
ZFHX3[1]	DN	S72A +/+<br>benign 0.002				S72A +/+<br>benign 0.002	
	R		S72A +/+<br>benign 0.002	S72A +/+<br>benign 0.002			S72A +/+<br>benign 0.002
	DN	V777A +/+	V777A +/+	V777A +/+	V777A +/+	V777A +/+	V777A +/+

	R	benign 0.006	benign 0.006	benign 0.006	benign 0.006	benign 0.006	benign 0.006
	DN<br>R		S3513G +/+<br>benign 0.000	S3513G +/+<br>benign 0.000	S3513G +/+<br>benign 0.000		
	DN<br>R			G3527dup +/+<br>disruptive inframe insertion	G3527dup +/-<br>disruptive inframe insertion		

**gene**		**11_TRAR**	**21_TRAR**	**28_TRAR**	**29_TRAR**	**17_TRAR**
ABL1						
ACTG1						
AK1 [1]						
AKT1 [2]						
AKT2 [3]	DN					A382T +/-<br>benign 0.001
	R					
AKT3 [2]						
ALK	DN	I1461V +/+<br>benign 0.000		I1461V +/+<br>benign 0.000	I1461V +/+<br>benign 0.000	
	R		I1461V +/-<br>benign 0.000			I1461V +/+<br>benign 0.000
AMER1	DN<br>R					T625A +/+<br>benign 0.000
ANKRD11 [1]	DN<br>R		D2376E +/+<br>benign 0.010	D2376E +/+<br>benign 0.010	D2376E +/+<br>benign 0.010	
	DN<br>R					P2263S +/-<br>benign 0.002
	DN<br>R					A2023P +/-<br>benign 0.451
APC	DN		V1822D +/-<br>benign 0.000	V1822D +/-<br>benign 0.000	V1822D +/-<br>benign 0.000	V1822D +/-<br>benign 0.000
	R					
ARAF						
ARF1						
ARID1A [1]						
ARID1B [1]						
ARID2 [4]						
ARID4A [1]	DN<br>R	N724S +/+<br>benign 0.003				N724S +/-<br>benign 0.003
	DN	T779A +/+				T779A +/-

	R	benign 0.000				benign 0.000
ARID4B[1]						
ARID5B[1]						
ASXL1	DN<br>R	G704R +/-<br>probably damaging 0.986				
	DN<br>R	L815P +/+<br>benign 0.000	L815P +/+<br>benign 0.000	L815P +/+<br>benign 0.000	L815P +/+<br>benign 0.000	L815P +/+<br>benign 0.000
ASXL2[1]	DN<br>R					K127R +/-<br>benign 0.003
ATM	DN<br>R	N1938S +/+<br>benign 0.000	N1938S +/+<br>benign 0.000	N1938S +/+<br>benign 0.000	N1938S +/+<br>benign 0.000	N1938S +/+<br>benign 0.000
ATR[1,4]	DN<br>R	R2425Q +/-<br>benign 0.000				
	DN<br>R	M211T +/+<br>benign 0.000	M211T +/-<br>benign 0.000	M211T +/-<br>benign 0.000	M211T +/-<br>benign 0.000	M211T +/-<br>benign 0.000
	DN<br>R	N1668fs +/-<br>frameshift variant				
ATRX[1]						
ATXN2[1]	DN<br>R	Q188 +/+<br>disruptive inframe deletion	Q188 +/+<br>disruptive inframe deletion	Q188 +/+<br>disruptive inframe deletion	Q188 +/+<br>disruptive inframe deletion	Q188 +/+<br>disruptive inframe deletion
	DN<br>R	L107V +/+<br>benign 0.000	L107V +/-<br>benign 0.000	L107V +/-<br>benign 0.000	L107V +/-<br>benign 0.000	L107V +/-<br>benign 0.000
AXIN1						
AXIN2	DN<br>R	P50S +/+<br>benign 0.000		P50S +/+<br>benign 0.000	P50S +/+<br>benign 0.000	P50S +/+<br>benign 0.000
AXL	DN<br>R	N266D +/+<br>benign 0.000	N266D +/+<br>benign 0.000	N266D +/+<br>benign 0.000	N266D +/+<br>benign 0.000	N266D +/+<br>benign 0.000
B2M[1]						
BACH2						
BAP1						
BCL2L11 (BIM)[1]						
BCL10[1]						
BCL11B	DN<br>R	V690M +/-<br>benign 0.082				
BCOR						
BCORL1[1]	DN<br>R	F111L +/+<br>benign 0.000	F111L +/+<br>benign 0.000	F111L +/+<br>benign 0.000	F111L +/+<br>benign 0.000	F111L +/+<br>benign 0.000
BLM[1]						

BMPR1A[1]	DN					P2T +/+<br>benign 0.000
	R					
BOP1						
BRAF[2, 5, 6]	DN	V600E +/+<br>probably damaging 0.971	V600E +/-<br>probably damaging 0.971	V600E +/-<br>probably damaging 0.971	V600E +/-<br>probably damaging 0.971	
	R					
BRCA1[1]	DN	S1634G +/-<br>benign 0.002				S1634G +/+<br>benign 0.002
	R					
	DN	K1183R +/-<br>benign 0.000				K1183R +/+<br>benign 0.000
	R					
	DN	E1038G +/-<br>benign 0.012				E1038G +/+<br>benign 0.012
	R					
	DN	P871L +/-<br>benign 0.000				P871L +/+<br>benign 0.000
	R					
BRCA2	DN	V2466A +/+<br>benign 0.000	V2466A +/+<br>benign 0.000	V2466A +/+<br>benign 0.000	V2466A +/+<br>benign 0.000	V2466A +/+<br>benign 0.000
	R					
	DN		T1915M +/-<br>benign 0.000	T1915M +/-<br>benign 0.000	T1915M +/-<br>benign 0.000	
	R		T1915M +/+<br>benign 0.000			
	DN					N372H +/-<br>benign 0.000
	R					
BRIP1[1]	DN	S919P +/+<br>benign 0.000	S919P +/-<br>benign 0.000	S919P +/+<br>benign 0.000	S919P +/+<br>benign 0.000	S919P +/-<br>benign 0.000
	R		S919P +/+<br>benign 0.000			
CALR[1]						
CARD11						
CBL						
CCNA1 (cyclin A1)						
CCNA2 (cyclin A2)	DN	I163V +/+<br>benign 0.000	I163V +/-<br>benign 0.000	I163V +/-<br>benign 0.000	I163V +/-<br>benign 0.000	I163V +/+<br>benign 0.000
	R					
CCNB1 (cyclin B1)						
CCNB2 (cyclin B2)						
CCND1 (cyclin D1)[7]						
CCND2 (cyclin D2)						
CCND3 (cyclin D3)	DN	S259A +/+<br>benign 0.000				S259A +/-<br>benign 0.000
	R					
CCNE1 (cyclin E1)						

CCNE2 (cyclin E2)						
CCNH (cyclin H)	DN<br>R		V270A +/-<br>probably damaging 0.965	V270A +/-<br>probably damaging 0.965	V270A +/-<br>probably damaging 0.965	
CDC25A						
CDC25B						
CDC73[1, 4]						
CD79B						
CDH1[1]						
CDK1						
CDK2						
CDK4						
CDK6						
CDK7						
CDK12[1]	DN					
	R	K804 +/-<br>inframe deletion				
CDKN1A (p21)						
CDKN1B (p27)	DN<br>R	V109G +/-<br>benign 0.047				V109G +/-<br>benign 0.047
CDKN2A (p16)[2]	DN<br>R	E69* +/+ stop gained<br>E61* +/+ stop gained				
CDKN2B (p15)						
CDKN2C (p18)						
CDKN2D (p19)						
CENPA[1]						
CHEK1 (Chk-1)[1]	DN<br>R	I471V +/+<br>benign 0.000	I471V +/+<br>benign 0.000	I471V +/+<br>benign 0.000	I471V +/+<br>benign 0.000	I471V +/+<br>benign 0.000
CHEK2 (Chk-2)						
CIC[1]						
CIITA	DN<br>R	R175G +/+<br>benign 0.000	R175G +/+<br>benign 0.000	R175G +/+<br>benign 0.000	R175G +/+<br>benign 0.000	R175G +/+<br>benign 0.000
	DN<br>R	Q901R +/+<br>benign 0.001	Q901R +/+<br>benign 0.001	Q901R +/+<br>benign 0.001	Q901R +/+<br>benign 0.001	Q901R +/+<br>benign 0.001
	DN<br>R		G501A +/+<br>benign 0.001	G501A +/+<br>benign 0.001	G501A +/+<br>benign 0.001	
CRBN						
CREB						

CREBBP[1]	DN	V1650G +/- benign 0.183				
	R					
CRLF2						
CTCF[4]						
CTLA4	DN		T17A +/- benign 0.015	T17A +/- benign 0.015	T17A +/- benign 0.015	T17A +/- benign 0.015
	R					
CTNNB1 (β-catenin)						
CTR9						
CUX1[1]						
CXCL8 (IL-8)						
CXCR1						
CXCR2						
DAXX[1]						
DDX3X[1]						
DDX53	DN	M381I +/+ probably damaging 1.000	M381I +/- probably damaging 1.000			
	R					
	DN	R391M +/+ benign 0.000	R391M +/- benign 0.000			
	R					
DEK	DN		S681P +/- benign 0.000		S681P +/- benign 0.000	
	R					
DICER1[1,4]						
DNMT3A						
DUSP1 (MKP1)						
DUSP4 (MKP2)[8]						
DUSP6 (MKP3)	DN	V114L +/- benign 0.075	V114L +/+ benign 0.075	V114L +/+ benign 0.075	V114L +/+ benign 0.075	V114L +/+ benign 0.075
	R					
DUSP7 (MKPX)						
DUSP10 (MKP5)						
DUSP14 (MKP6)						
DUSP16 (MKP7)	DN					
	R	V366M +/- benign 0.005				
DUSP22						
E2F1	DN					G393S +/- benign 0.022
	R					
E2F3	DN	D148N +/- possibly damaging 0.915 D389N +/-				
	R					

		benign 0.017				
ECT2L	DN	K676fs +/- frameshift variant				
	R					
	DN					E527K +/- benign 0.002
	R					
EED						
EGFR	DN	R521K +/- benign 0.000	R521K +/- benign 0.000	R521K +/- benign 0.000	R521K +/- benign 0.000	R521K +/- benign 0.000
	R					
EIF1AX[1]						
ELF3	DN					D91N +/- probably damaging 1.000
	R					
ELK1						
EP300[1]	DN	I997V +/+ benign 0.000				I997V +/- benign 0.000
	R					
	DN	Q2223P +/- benign 0.000				
	R					
EP400[1]	DN		A574V +/- possibly damaging 0.854	A574V +/- possibly damaging 0.854	A574V +/- possibly damaging 0.854	
	R					
	DN		A3094T +/- benign 0.001	A3094T +/- benign 0.001	A3094T +/- benign 0.001	
	R					
	DN		Q2742 +/- disruptive inframe insertion	Q2742 +/- disruptive inframe insertion	Q2742 +/- disruptive inframe insertion	
	R					
EPHA3[1]	DN	W924R +/- benign 0.000	W924R +/- benign 0.000		W924R +/- benign 0.000	W924R +/+ benign 0.000
	R			W924R +/- benign 0.000		
EPHA7[1]						
EPHB1[1]						
ERBB2	DN		P8T +/- benign 0.000	P8T +/+ benign 0.000	P8T +/+ benign 0.000	
	R		P8T +/+ benign 0.000			
	DN					
	R					I655V +/- benign 0.406
	DN	P1170A +/- possibly damaging 0.953	P1170A +/+ possibly damaging 0.953	P1170A +/+ possibly damaging 0.953	P1170A +/+ possibly damaging 0.953	P1170A +/- possibly damaging 0.953
	R					
ERBB3						

ERBB4	DN					
	R	L437F +/- possibly damaging 0.928 K438M +/- possibly damaging 0.894				
ERCC2	DN		D312N +/- benign 0.065	D312N +/- benign 0.065	D312N +/- benign 0.065	
	R					
	DN		K751Q +/- benign 0.000	K751Q +/- benign 0.000	K751Q +/- benign 0.000	
	R					
ERF						
ERRFI1						
ESCO2[1]						
ETS2[3]	DN	A19T +/- ?				
	R					
ETV6						
EZH2	DN				S412C +/- probably damaging 1.000	
	R					
FAM58A[1]	DN	A18G +/+ frameshift variant				
	R					
	DN	A6G +/+ frameshift variant				
	R					
FANCA[1]	DN	T266A +/+ benign 0.000				T266A +/- benign 0.000
	R					
	DN					G809D +/- benign 0.000
	R					
	DN					G501S +/- benign 0.000
	R					
FANCD2[1]	DN	N405S +/- benign 0.022	N405S +/- benign 0.022	N405S +/- benign 0.022	N405S +/- benign 0.022	
	R					
FAS[1]						
FAT1[1]	DN	K4059N +/+ benign 0.000	K4059N +/+ benign 0.000	K4059N +/+ benign 0.000	K4059N +/+ benign 0.000	K4059N +/+ benign 0.000
	R					
	DN	Q2933P +/+ benign 0.000	Q2933P +/+ benign 0.000	Q2933P +/+ benign 0.000	Q2933P +/+ benign 0.000	Q2933P +/+ benign 0.000
	R					
	DN	R1064G +/+ benign 0.000	R1064G +/+ benign 0.000	R1064G +/+ benign 0.000	R1064G +/+ benign 0.000	R1064G +/+ benign 0.000
	R					
	DN	V862L +/+ benign 0.000	V862L +/+ benign 0.000	V862L +/+ benign 0.000	V862L +/+ benign 0.000	V862L +/+ benign 0.000
	R					

	DN	F614L +/+<br>benign 0.000	F614L +/+<br>benign 0.000	F614L +/+<br>benign 0.000	F614L +/+<br>benign 0.000	F614L +/+<br>benign 0.000
	R					
	DN	S404R +/+<br>benign 0.032	S404R +/+<br>benign 0.032	S404R +/+<br>benign 0.032	S404R +/+<br>benign 0.032	S404R +/+<br>benign 0.032
	R					
	DN	V482I +/+<br>benign 0.009	V482I +/+<br>benign 0.009	V482I +/+<br>benign 0.009	V482I +/+<br>benign 0.009	V482I +/+<br>benign 0.009
	R					
	DN	H1273R +/+<br>benign 0.000				H1273R +/+<br>benign 0.000
	R					
FBXO11						
FBXW7[1]	DN	V418M +/+<br>possibly damaging 0.801				
	R					
FGFR1						
FGFR2	DN	E637K +/+<br>probably damaging 0.970				
	R					
FGFR3	DN		P451S +/-<br>possibly damaging 0.902	P451S +/-<br>possibly damaging 0.902	P451S +/-<br>possibly damaging 0.902	
	R					
FGFR4	DN			G388R +/-<br>probably damaging 0.998	G388R +/-<br>probably damaging 0.998	G388R +/+<br>probably damaging 0.998
	R		G388R +/-<br>probably damaging 0.998			
	DN	P136L +/-<br>benign 0.000<br>V10I +/-<br>benign 0.000	P136L +/-<br>benign 0.000	P136L +/-<br>benign 0.000	P136L +/-<br>benign 0.000	P136L +/+<br>benign 0.000<br>V10I +/-<br>benign 0.000
	R					
FH						
FLT3	DN					
	R					D7G +/+<br>benign 0.000
	DN	T227M +/+<br>probably damaging 0.999				T227M +/+<br>probably damaging 0.999
	R					
FOXA1	DN					S448N +/-<br>benign 0.001
	R					S448N +/+<br>benign 0.001
FOXM1	DN					
	R		S681P +/-<br>benign 0.000	S681P +/-<br>benign 0.000	S681P +/-<br>benign 0.000	
FOXO3A						
FOXP1[1]						
GADD45A						

GADD45B						
GADD45G						
GNA11						
GNAS[1, 4]	DN					
	R					
	DN	A436D +/-<br>benign 0.001				
	R					
GNB1[1]						
GRB2						
GRIN2A[1, 4]						
GSK3A						
GSK3B						
HDAC4						
HIST1H2BC[1]						
HIST1H3H[1]						
HLA-A[1]						
HLA-B						
HNF1A	DN	S574G +/+<br>benign 0.000	S574G +/+<br>benign 0.000	S574G +/+<br>benign 0.000	S574G +/+<br>benign 0.000	S574G +/+<br>benign 0.000
	R					
	DN		I27L +/-<br>benign 0.002	I27L +/-<br>benign 0.002	I27L +/-<br>benign 0.002	
	R					
	DN		S487N +/-<br>benign 0.000	S487N +/-<br>benign 0.000	S487N +/-<br>benign 0.000	
	R					
	DN					A98V +/-<br>benign 0.209
	R					
HOXD8[9]						
HRAS	DN					Q61R +/-<br>benign 0.008
	R					
IDH1[4]						
IDH2[4]						
IGF1R						
IGFBP7	DN					
	R	L11F +/-<br>benign 0.005	L11F +/-<br>benign 0.005		L11F +/-<br>benign 0.005	
INPP4B						
INPPL1	DN	L632I +/-<br>probably damaging 0.990				
	R					
	DN	A1083G +/-				

	R	benign 0.000				
IRF8 [1]						
JAK1 [1]						
JAK2						
JARID2 [1]						
KDM5C						
KDM6A	DN	T778K +/+<br>benign 0.000				T778K +/+<br>benign 0.000
	R					
KDR (VEGFR2)[4]	DN		Q472H +/-<br>benign 0.003	Q472H +/-<br>benign 0.003	Q472H +/-<br>benign 0.003	
	R					
	DN		V297I +/-<br>probably damaging 1.000	V297I +/-<br>probably damaging 1.000	V297I +/-<br>probably damaging 1.000	
	R					
KEAP1						
KIT						
KMT2A[1]	DN		A30G +/+<br>possibly damaging 0.953	A30G +/+<br>possibly damaging 0.953	A30G +/+<br>possibly damaging 0.953	
	R					
	DN					R2191* +/+<br>stop gained
	R					
KMT2B[1]	DN	G296S +/-<br>probably damaging 1.000				
	R		G296S +/+<br>probably damaging 1.000	G296S +/-<br>probably damaging 1.000		
	DN	D2364G +/-<br>benign 0.000	D2364G +/-<br>benign 0.000	D2364G +/-<br>benign 0.000	D2364G +/-<br>benign 0.000	D2364G +/+<br>benign 0.000
	R					
	DN	R1021fs +/+<br>frameshift variant	R1021fs +/+<br>frameshift variant	R1021fs +/+<br>frameshift variant	R1021fs +/+<br>frameshift variant	R1021fs +/+<br>frameshift variant
	R					
	DN					P1829L +/-<br>benign 0.006
	R					
KMT2C[1,4]						
KMT2D [1, 4]	DN	V4305I +/+<br>benign 0.039				
	R					
	DN		P813L +/-<br>benign 0.000	P813L +/-<br>benign 0.000	P813L +/-<br>benign 0.000	
	R					
KNSTRN	DN	N279Y +/-<br>probably damaging 0.965				
	R					
KRAS[2, 3, 10]						
LAMTOR1 (MP1)						
LATS1[1]						
MAP2K1 (MEK1) [9, 11-13]	DN					P124S +/-

	R					probably damaging 0.999
MAP2K2 (MEK2) [3, 9, 14]	DN					
	R				L201V +/-<br>probably damaging 1.000<br>F57V +/-<br>probably damaging 0.991	
MAP2K4 [1]						
MAPK3 (ERK1)						
MAPK1 (ERK2)						
MAPK8 (JNK1)						
MAPK9 (JNK2)						
MAPK10 (JNK3)						
MAPK14 (p38)						
MC1R	DN	R151C +/+<br>probably damaging 1.000				
	R					
	DN		V60L +/-<br>probably damaging 0.988			
	R					
	DN					R163Q +/-<br>benign 0.004
	R					
MDM2						
MDM4						
MED12 [1]	DN	H2116 +/+<br>disruptive inframe insertion				
	R					
MEN1 [4]	DN	T546A +/+<br>benign 0.000	T546A +/+<br>benign 0.000	T546A +/+<br>benign 0.000	T546A +/+<br>benign 0.000	T546A +/+<br>benign 0.000
	R					
MET						
MGA [1]	DN	T716S +/-<br>benign 0.000	T716S +/+<br>benign 0.000	T716S +/+<br>benign 0.000	T716S +/+<br>benign 0.000	T716S +/+<br>benign 0.000
	R					
	DN		P1523A +/+<br>possibly damaging 0.657	P1523A +/+<br>possibly damaging 0.657	P1523A +/+<br>possibly damaging 0.657	
	R					
MITF [9]						
MKI67 (Ki67)	DN	I2101T +/+<br>benign 0.008	I2101T +/-<br>benign 0.008	I2101T +/-<br>benign 0.008	I2101T +/-<br>benign 0.008	I2101T +/-<br>benign 0.008
	R		I2101T +/+<br>benign 0.008			I2101T +/+<br>benign 0.008

	DN	N104S +/+<br>probably damaging 0.982	N104S +/+<br>probably damaging 0.982<br>T2868S +/-<br>probably damaging 0.999<br>R2786Q +/-<br>probably damaging 0.991<br>E1403V +/-<br>probably damaging 0.997<br>K3217E +/-<br>benign 0.000<br>T3150S +/-<br>benign 0.000<br>N2363S +/-<br>benign 0.000<br>G1042S +/-<br>benign 0.401	N104S +/+<br>probably damaging 0.982<br>T2868S +/-<br>probably damaging 0.999<br>R2786Q +/-<br>probably damaging 0.991<br>E1403V +/-<br>probably damaging 0.997<br>K3217E +/-<br>benign 0.000<br>T3150S +/-<br>benign 0.000<br>N2363S +/-<br>benign 0.000<br>G1042S +/-<br>benign 0.401	N104S +/+<br>probably damaging 0.982<br>T2868S +/-<br>probably damaging 0.999<br>R2786Q +/-<br>probably damaging 0.991<br>E1403V +/-<br>probably damaging 0.997<br>K3217E +/-<br>benign 0.000<br>T3150S +/-<br>benign 0.000<br>N2363S +/-<br>benign 0.000<br>G1042S +/-<br>benign 0.401	N104S +/+<br>probably damaging 0.982<br>T2868S +/-<br>probably damaging 0.999<br>R2786Q +/-<br>probably damaging 0.991<br>E1403V +/-<br>probably damaging 0.997<br>K3217E +/-<br>benign 0.000<br>T3150S +/-<br>benign 0.000<br>N2363S +/-<br>benign 0.000<br>G1042S +/-<br>benign 0.401
	R					
MLH1[1]	DN		I219V +/-<br>benign 0.018			
	R					
MMP2						
MOS	DN	D201V +/+<br>probably damaging 1.000				
	R					
MPL[1]						
MRE11A[1]						
MSH2						
MSH3[1]	DN	Q949R +/+<br>benign 0.000	Q949R +/+<br>benign 0.000	Q949R +/+<br>benign 0.000	Q949R +/+<br>benign 0.000	Q949R +/-<br>benign 0.000
	R					
	DN	A1045T +/-<br>benign 0.075	A1045T +/-<br>benign 0.075	A1045T +/-<br>benign 0.075	A1045T +/-<br>benign 0.075	A1045T +/-<br>benign 0.075
	R					
	DN	A61-P63dup +/-<br>inframe insertion	A61-P63dup +/-<br>inframe insertion	A61-P63dup +/-<br>inframe insertion	A61-P63dup +/-<br>inframe insertion	A61-P63dup +/-<br>inframe insertion
	R					
	DN					I79V +/+<br>benign 0.000
	R					
MSH6[1]	DN		G39E +/-<br>benign 0.000	G39E +/-<br>benign 0.000	G39E +/-<br>benign 0.000	G39E +/-<br>benign 0.000
	R					
MTOR	DN	R2152C +/-<br>benign 0.050				
	R					
MUTYH[1]	DN	Q338H +/-<br>benign 0.343	Q338H +/+<br>benign 0.343	Q338H +/+<br>benign 0.343	Q338H +/+<br>benign 0.343	
	R					
	DN					V22M +/-

	R					benign 0.185
MYC						
MYD88[1]						
MYT1	DN		T782S +/-<br>benign 0.035	T782S +/-<br>benign 0.035	T782S +/-<br>benign 0.035	
	R					
NBN [1]	DN	E185Q +/-<br>benign 0.001				
	R					
NCOR1[1]						
NF1[15]	DN			R135W +/-<br>probably damaging 1.000		
	R					
NF2[1]						
NFKBIA						
NGFR						
NOS3 (eNOS)	DN	D298E +/+<br>benign 0.000	D298E +/-<br>benign 0.000	D298E +/-<br>benign 0.000	D298E +/-<br>benign 0.000	D298E +/+<br>benign 0.000
	R					
NOTCH1[1]						
NOTCH2[1]	DN	F1209V +/-<br>possibly damaging 0.939				
	R					
	DN					
	R	N46S +/-<br>benign 0.010		N46S +/-<br>benign 0.010		N46S +/-<br>benign 0.010
	DN					
	R	E38K +/-<br>benign 0.044		E38K +/-<br>benign 0.044		E38K +/-<br>benign 0.044
	DN					
	R	C19W +/-<br>benign 0.001	C19W +/-<br>benign 0.001	C19W +/-<br>benign 0.001	C19W +/-<br>benign 0.001	C19W +/-<br>benign 0.001
	DN					
	R	P6fs +/-<br>frameshift variant	P6fs +/-<br>frameshift variant	P6fs +/-<br>frameshift variant	P6fs +/-<br>frameshift variant	P6fs +/-<br>frameshift variant
	DN					
	R		A21T +/-<br>benign 0.000	A21T +/-<br>benign 0.000		
NOTCH3	DN	C1826F +/-<br>possibly damaging 0.829				
	R					
	DN	A2223V +/+<br>benign 0.001	A2223V +/+<br>benign 0.001	A2223V +/+<br>benign 0.001	A2223V +/+<br>benign 0.001	A2223V +/+<br>benign 0.001
	R					
NOTCH4[4]						

NRAS[2, 12]						
NSD1[1]	DN					S726P +/+<br>probably damaging 0.999
	R					
	DN					V614L +/+<br>benign 0.002
	R					
NTRK1						
NTRK2						
NTRK3						
PAK5[1]						
PALB2[1]						
PARK2[1]						
PARP1[1]						
PAX5[1]	DN	G266E +/+<br>benign 0.000				
	R					
	DN		T293I +/+<br>benign 0.000	T293I +/+<br>benign 0.000	T293I +/+<br>benign 0.000	T293I +/+<br>benign 0.000
	R					
PBRM1[1, 4]						
PDGFRA	DN					
	R		E36A +/-<br>probably damaging 1.000			
PDGFRB						
PDPK1 (PDK1)						
PHLPP1[2]						
PIGA[1]						
PIK3CA[2]	DN					
	R		G1049R +/-<br>benign 0.300			
PIK3CB						
PIK3CG[2]						
PIK3R1						
PIK3R2[2]	DN	S313P +/+<br>benign 0.000<br>S234R +/+<br>benign 0.000	S313P +/+<br>benign 0.000<br>S234R +/+<br>benign 0.000	S313P +/+<br>benign 0.000<br>S234R +/+<br>benign 0.000	S313P +/+<br>benign 0.000<br>S234R +/+<br>benign 0.000	S313P +/+<br>benign 0.000<br>S234R +/+<br>benign 0.000
	R					
PIK3R3	DN					
	R	N283K +/+<br>benign 0.000	N283K +/-<br>benign 0.000	N283K +/-<br>benign 0.000	N283K +/-<br>benign 0.000	N283K +/+<br>benign 0.000
PMS1[1]						
PMS2[1]	DN	K541E +/+	K541E +/-	K541E +/-	K541E +/-	K541E +/-

	R	benign 0.000	benign 0.000	benign 0.000	benign 0.000	benign 0.000
	DN<br>R	P470S +/-<br>benign 0.018				
	DN<br>R					R20Q +/-<br>possibly damaging 0.857
POT1						
PPP2R1A						
PPP6C [1]						
PRDM1[1]	DN<br>R	G74S +/-<br>benign 0.016				
	DN<br>R					I117M +/-<br>possibly damaging 0.950
	DN<br>R					D203E +/-<br>possibly damaging 0.660
PRKAA1 (AMPK1)						
PRKAA2 (AMPK2)						
PRKACA (PKA)						
PRKACB (PKA)						
PTCH1	DN<br>R		P1315L +/-<br>possibly damaging 0.944	P1315L +/-<br>possibly damaging 0.944	P1315L +/-<br>possibly damaging 0.944	
PTEN[2]						
PTK2 (FAK)						
PTPN1						
PTPN11						
PTPRD [1, 4]	DN<br>R	G272R +/-<br>probably damaging 0.977				
	DN<br>R		T781A +/-<br>benign 0.007	T781A +/-<br>benign 0.007	T781A +/-<br>benign 0.007	
PTPRS	DN<br>R	C1457R +/+<br>benign 0.000	C1457R +/+<br>benign 0.000	C1457R +/+<br>benign 0.000	C1457R +/+<br>benign 0.000	C1457R +/+<br>benign 0.000
PTPRT[4]	DN<br>R					A29P +/+<br>benign 0.014
RAC1[9, 16]						
RAD50 [1]						
RAD51						
RAF1 (CRAF)						
RASA2						

RASGRF1	DN<br>R					S752N +/-<br>benign 0.001
RASGRF2	DN<br>R	S753P +/+<br>benign 0.002				
RASGRP1						
RASGRP2						
RASGRP3	DN<br>R	T393A +/-<br>benign 0.131				
RASGRP4	DN<br>R	H311Y +/-<br>probably damaging 0.993<br>G165R +/-<br>benign 0.001<br>I18T +/-<br>benign 0.000				E519K +/-<br>probably damaging 0.997
RASSF2						
RB1						
RBM10[17]	DN<br>R					R149Q +/+<br>possibly damaging 0.928
RBMX [17]	DN					
	R		G379R +/-<br>probably damaging 1.000<br>Y357H +/-<br>probably damaging 0.995<br>R339G +/-<br>possibly damaging 0.995<br>S337N +/-<br>probably damaging 0.981<br>R324P +/-<br>probably damaging 0.996<br>S303fs +/-<br>frameshift variant<br>P301L +/-<br>benign 0.269	G379R +/-<br>probably damaging 1.000<br>Y357H +/-<br>probably damaging 0.995<br>R339G +/-<br>possibly damaging 0.995<br>S337N +/-<br>probably damaging 0.981<br>R324P +/-<br>probably damaging 0.996<br>S303fs +/-<br>frameshift variant<br>P301L +/-<br>benign 0.269	G379R +/-<br>probably damaging 1.000<br>Y357H +/-<br>probably damaging 0.995<br>R339G +/-<br>possibly damaging 0.995<br>S337N +/-<br>probably damaging 0.981<br>R324P +/-<br>probably damaging 0.996	
RECQL[1]	DN<br>R					D481fs +/-<br>frameshift variant
RET	DN<br>R	G691S +/+<br>benign 0.062				
RHEB						
RICTOR	DN<br>R		S837F +/+<br>benign 0.002	S837F +/+<br>benign 0.002	S837F +/+<br>benign 0.002	S837F +/-<br>benign 0.002

RIT1						
RNF43[4]	DN<br>R	P231L +/+<br>benign 0.056				
	DN<br>R		I47V +/-<br>benign 0.002	I47V +/+<br>benign 0.002	I47V +/+<br>benign 0.002	I47V +/-<br>benign 0.002
	DN<br>R		L418M +/-<br>probably damaging 0.969	L418M +/+<br>probably damaging 0.969	L418M +/+<br>probably damaging 0.969	L418M +/-<br>probably damaging 0.969
	DN<br>R		R343H +/-<br>benign 0.002	R343H +/-<br>benign 0.002	R343H +/-<br>benign 0.002	R343H +/-<br>benign 0.002
	DN<br>R					
ROS1	DN<br>R	D2213N +/-<br>benign 0.009<br>S2229C +/-<br>benign 0.000<br>K2228Q +/-<br>benign 0.000	D2213N +/+<br>benign 0.009<br>S2229C +/+<br>benign 0.000<br>K2228Q +/+<br>benign 0.000	D2213N +/+<br>benign 0.009<br>S2229C +/+<br>benign 0.000<br>K2228Q +/+<br>benign 0.000	D2213N +/+<br>benign 0.009<br>S2229C +/+<br>benign 0.000<br>K2228Q +/+<br>benign 0.000	D2213N +/-<br>benign 0.009<br>S2229C +/-<br>benign 0.000<br>K2228Q +/-<br>benign 0.000<br>S1109L +/-<br>benign 0.014
RPS6KA1 (RSK1)	DN	K344T +/-<br>benign 0.088				
	R	K344T +/+<br>benign 0.088				
RPS6KA2 (RSK3)	DN<br>R	T34A +/+<br>benign 0.000<br>E32G +/+<br>benign 0.000	T34A +/+<br>benign 0.000<br>E32G +/+<br>benign 0.000	T34A +/+<br>benign 0.000<br>E32G +/+<br>benign 0.000	T34A +/+<br>benign 0.000<br>E32G +/+<br>benign 0.000	T34A +/+<br>benign 0.000<br>E32G +/+<br>benign 0.000
RPS6KA3 (RSK2)						
RPS6KA4 (MSK2)	DN<br>R	S758A +/-<br>benign 0.000	S758A +/-<br>benign 0.000	S758A +/-<br>benign 0.000		S758A +/+<br>benign 0.000
RPS6KA5 (MSK1)						
RPS6KB1 (p70S6K)						
RPTOR	DN<br>R					A523V +/-<br>possibly damaging 0.745
RTEL1	DN<br>R	Q1042H +/-<br>benign 0.000	Q1042H +/+<br>benign 0.000	Q1042H +/+<br>benign 0.000	Q1042H +/+<br>benign 0.000	Q1042H +/+<br>benign 0.000
SAMD4B [3]						
SDHA[1]						
SDHB[4]						
SDHC[1]						

SDHD	DN<br>R					G12S +/+<br>benign 0.005
SETD2[1, 4]	DN<br>R		P1962L +/-<br>benign 0.001			P1962L +/-<br>benign 0.001
SF3B1[4]						
SH2D1A						
SHQ1	DN<br>R	S489N +/+<br>benign 0.000	S489N +/-<br>benign 0.000	S489N +/-<br>benign 0.000	S489N +/-<br>benign 0.000	
	DN<br>R	F72C +/-<br>probably damaging 0.971				
SLX4	DN<br>R					R204C +/-<br>probably damaging 1.000
	DN<br>R					E248K +/-<br>possibly damaging 0.798
	DN<br>R					M386V +/-<br>benign 0.000
	DN<br>R					N457K +/-<br>benign 0.000
	DN<br>R					L671S +/-<br>benign 0.000
	DN<br>R					A952T +/-<br>benign 0.056
	DN<br>R					A952V +/-<br>possibly damaging 0.926
	DN<br>R					P1122L +/-<br>benign 0.003
	DN<br>R					A1221V +/-<br>benign 0.019
SMAD3						
SMAD4[1]						
SMARCA4 (BRG1)[1]						
SOS1	DN<br>R	R647M +/-<br>probably damaging 0.998				
SOS2						
SOX2	DN					
	R					T222I +/-

						possibly damaging 0.804
SOX9						
SOX10						
SPEN[1]	DN	A970V +/- benign 0.006				
	R					
	DN	L1091P +/+ benign 0.000				L1091P +/- benign 0.000
	R					
	DN	N2360D +/+ benign 0.000				N2360D +/- benign 0.000
	R					
SPOP[1]						
SPRED1						
SRC						
STAG2[1]						
STAT3						
STAT5A						
STAT5B						
STK11						
STK19						
SUFU						
SYK						
TBX2						
TCF3[1]						
TCF7L2[1]						
TERT						
TET1	DN	V128F +/+ possibly damaging 0.845				
	R					
	DN		D162G +/- benign 0.295	D162G +/- benign 0.295	D162G +/- benign 0.295	
	R					
	DN	I1123M +/+ benign 0.070	I1123M +/+ benign 0.070	I1123M +/+ benign 0.070	I1123M +/+ benign 0.070	I1123M +/- benign 0.070
	R					
	DN					S193T +/+ benign 0.107
	R					
TET2[4]	DN	I1762V +/+ benign 0.001	I1762V +/- benign 0.001	I1762V +/- benign 0.001	I1762V +/- benign 0.001	I1762V +/- benign 0.001
	R					
	DN					L34F +/- benign 0.246
	R					
	DN					V218M +/-

	R					benign 0.000
	DN					M1701I +/-<br>benign 0.001
	R					
	DN					H1778R +/-<br>probably damaging 0.994
	R					
TGFBR2						
TMEM127[1]						
TOP1	DN	H81Y +/-<br>benign 0.273				
	R					
TP53	DN	P72R +/-<br>benign 0.083	P72R +/-<br>benign 0.083	P72R +/+<br>benign 0.083	P72R +/+<br>benign 0.083	P72R +/-<br>benign 0.083
	R		P72R +/+<br>benign 0.083			P72R +/+<br>benign 0.083
	DN					
	R					R156H +/-<br>probably damaging 1.000
TP53BP1[1]	DN	K1141Q +/-<br>benign 0.000				K1141Q +/-<br>benign 0.000
	R					
	DN	G417S +/-<br>benign 0.000				G417S +/-<br>benign 0.000
	R					
	DN	D358E +/-<br>benign 0.000				D358E +/-<br>benign 0.000
	R					
TP63[4]						
TSC1	DN		M322T +/-<br>benign 0.000	M322T +/-<br>benign 0.000	M322T +/-<br>benign 0.000	
	R					
TSC2	DN					
	R			D1004Y +/-<br>probably damaging 1.000		
TYRO3	DN	I346N +/-<br>benign 0.408<br>V669L +/-<br>probably damaging 1.000				I346N +/-<br>benign 0.408
	R					
VHL						
WEE1						
WNK1	DN	T1316P +/-<br>benign 0.000<br>C1766S +/+<br>benign 0.000	T1316P +/+<br>benign 0.000<br>C1766S +/+<br>benign 0.000<br>M2068I +/-<br>benign 0.000	T1316P +/+<br>benign 0.000<br>C1766S +/+<br>benign 0.000<br>M2068I +/-<br>benign 0.000	T1316P +/+<br>benign 0.000<br>C1766S +/+<br>benign 0.000<br>M2068I +/-<br>benign 0.000	T1316P +/+<br>benign 0.000<br>C1766S +/+<br>benign 0.000
	R					

WT1						
XRCC2[1]						
ZFHX3[1]	DN	S72A +/+ benign 0.002				
	R					
	DN	V777A +/+ benign 0.006				V777A +/- benign 0.006
	R		V777A +/+ benign 0.006	V777A +/+ benign 0.006		
	DN					G3527dup +/- disruptive inframe insertion
	R					
	DN					A62V +/+ benign 0.147
	R					
	DN					A997S +/- probably damaging 0.999
	R					

References:

1. Skin Cutaneous Melanoma (Broad, Cancer Discov 2014); https://cbioportal.org; (**AK1** T278Qfs*15; **ANKRD11** D1104Mfs*214; **ARID1A** D1850Tfs*33; **ARID1A** D1850Tfs*33; **ARID1A** E1956Gfs*6; **ARID1A** Q567Sfs*52; **ARID1A** S547Qfs*76; **ARID1B** Q1183Sfs*15; **ARID1B** Y1322Tfs*113; **ARID4A** S1067Qfs*10; **ARID4A** S1067Rfs*17; **ARID4B** X92_splice; **ARID5B** K1027Rfs*8; **ASXL2** C1410*; **ATR** P1259Rfs*18; **ATR** X1274_splice; **ATR** X1913_splice; **ATRX** X1567_splice; **ATXN2** H1242Tfs*25; **BCL10** I46Yfs*24; **BCL2L11** X132_splice; **BCORL1** P1681Qfs*20; **BCORL1** A207Cfs*65; **BLM** D757Tfs*4; **BMPR1A** X144_splice; **BRCA1** K339Rfs*2; **BRCA1** Q1467*; **BRCA1** R1751*; **BRIP1** K345Rfs*3; **B2M** L15Ffs*41;**CALR** E405*; **CDC73** R229Sfs*37; **CDH1** R224C; **CDK12** G1271Dfs*23; **CDK12** Q1291Rfs*3; **CHEK2 (Chk-2)** K373E; **CENPA** R130Q; **CIC** X255_splice; **CREBBP** I1084Sfs*15; **CUX1** P929Rfs*2; **CUX1** X484_splice; **CUX1** X688_splice; **DAXX** E457del; **DAXX** X417_splice; **DAXX** Y344*; **DDX3X** K452Rfs*44; **DICER1** K1601Rfs*19;**EIF1AX** G8R; **EIF1AX** G8V; **EP300** X1224_splice; **EP400** A605Hfs*41; **EP400** F1094Lfs*4; **EP400** H31Pfs*34; **EP400** R2668*; **EPHA3** W826*; **EPHA7** W931*; **EPHB1** R743Q; **ESCO2** T254Nfs*27; **FAM58A** X97_splice; **FANCA** M262Cfs*13; **FANCD2** F925Lfs*6; **FANCD2** X1321_splice; **FAS** C135Vfs*52; **FAT1** A4444Qfs*43; **FAT1** L2345Sfs*16; **FAT1** R1627Q; **FBXW7** S582L; **FBXW7** S668Vfs*39; **FOXP1** L414*; **GNAS** R201H; **GNB1** R214*; **GRIN2A** P1132L; **GRIN2A** X443_splice; **HIST1H2BC** E72K; **HIST1H3H** R9C; **HLA-A** T206fs; **IRF8** S20Rfs*7; **JAK1** E371*; **JAK1** R174*; **JARID2** F659Sfs*3; **JARID2** X224_splice; **KMT2A** P773Rfs*8; **KMT2A** R862*; **KMT2A** S774Vfs*12; **KMT2B** R965*; **KMT2C** K3870Rfs*19; **KMT2C** K3870Rfs*19; **KMT2C** N2842Mfs*21; **KMT2C** R4478*; **KMT2C** W491*; **KMT2C** X1598_splice; **KMT2C** X4047_splice; **KMT2D** P647Hfs*283; **LATS1** R886*; **MAP2K4** R154L; **MAP2K4** A279T; **MED12** A3T; **MED12** C514*; **MED12** Q2076*; **MED12** R764*; **MGA** Q2477*; **MLH1** Q544*; **MLH1** R497Gfs*11; **MPL** L513del; **MRE11A** X182_splice; **MSH3** E261Gfs*43; **MSH3** K383Rfs*32; **MSH6** R732*; **MSH6** T1219I; **MSH6** W142*; **MSH6** X87_splice; **MUTYH** K518Sfs*50; **MYD88** P166S; **NBN** R466Gfs*18; **NBN** R551Gfs*8; **NCOR1** X244_splice; **NF2** T230Hfs*16; **NF2** X200_splice; **NOTCH1** A2052Efs*215; **NOTCH1** C1549*; **NOTCH1** G310E; **NOTCH1** X289_splice; **NOTCH2** A2333Gfs*66; **NOTCH2** E568*; **NOTCH2** P6Rfs*27; **NOTCH2** S1419Afs*8; **NOTCH2** X1771_splice; **NSD1** A2268Lfs*26; **NSD1** N576Tfs*23; **NSD1** R1605Gfs*37; **PAK5** M173I; **PALB2** X838_splice; **PARK2** N428Mfs*7; **PARP1** K700Sfs*2; **PARP1** P359Qfs*22; **PAX5** F27Lfs*2; **PBRM1** R710*; **PIGA** R412*; **PMS1** Q713*; **PMS2** X84_splice; **PPP6C** R264C; **PRDM1** L83Yfs*19; **PRDM1** Y267Pfs*3; **PTPRD** A420V; **PTPRD** Q1874*; **RAD50** N795Ifs*6; **RECQL** X556_splice; **SDHA** L649Efs*4; **SDHC** P64Hfs*33; **SETD2** R1598*; **SETD2** R620*; **SMAD4** G231Afs*10; **SMARCA4** L434Sfs*5; **SMARCA4** X1057_splice; **SPEN** P3317Rfs*83; **SPEN** R806Tfs*14; **SPEN** T3246Hfs*21; **SPOP** E253*;

STAG2 E28Kfs*43; **TCF3** Q361Rfs*33; **TCF7L2** C486Vfs*8; **TMEM127** Q231*; **TP53BP1** Q644*; **TP53BP1** P766Qfs*3; **XRCC2** L117Wfs*17; **ZFHX3** P3569Qfs*13; **ZFHX3** R1309*)

2. Shi, H.; Hugo, W.; Kong, X.; Hong, A.; Koya, R.; Moriceau, G.; Chodon, T.; Guo, R.; Johnson, D.B.; Dahlman, K.B.; Kelley, M.C.; Kefford, R.F.; Chmielowski, B.; Glaspy, J.A.; Sosman, J.A.; van Baren, N.; Long, G.V.; Ribas, A.; Lo, R.S. Acquired resistance and clonal evolution in melanoma during BRAF inhibitor therapy. *Cancer Discov*. **2014**, *4*, 80-93. doi: 10.1158/2159-8290.CD-13-0642 (**AKT1** Q79K; **AKT2** E17K; **BRAF** gene amplification splice variants; **CDKN2 A** deletion; **KRAS** Q61H; **KRAS** G12C/R; **NRAS** G12D/R; **NRAS** G13R; **NRAS** Q61K/R/L; **PHLPP1** K596E; **PIK3CA** D350G; **PIK3CA** E545G; **PIK3CG** V983E; **PIK3R2** N561D; **PTEN** M134del terminating frameshift; **PTEN** K128T)

3. Wagle, N.; Van Allen, E.M.; Treacy, D.J.; Frederick, D.T.; Cooper, Z.A.; Taylor-Weiner, A.; Rosenberg, M.; Goetz, E.M.; Sullivan, R.J.; Farlow, D.N.; Friedrich, D.C.; Anderka, K.; Perrin, D.; Johannessen, C.M.; McKenna, A.; Cibulskis, K.; Kryukov, G.; Hodis, E.; Lawrence, D.P.; Fisher, S.; Getz, G.; Gabriel, S.B.; Carter, S.L.; Flaherty, K.T.; Wargo, J.A.; Garraway, L.A. MAP kinase pathway alterations in BRAF-mutant melanoma patients with acquired resistance to combined RAF/MEK inhibition. *Cancer Discov*. **2014,** *4*, 61-68. doi: 10.1158/2159-8290.CD-13-0631 (**AKT2** gene amplification; **ETS2** P53S; **KRAS** Q61H; **KRAS** G12C/R; **MAP2K2** C125S; **MAP2K2** L46F; **MAP2K2** N126D; **MAP2K2** Q60P; **MAP2K2** V35M; **SAMD4B** gene amplification)

4. MSKCC, JCO Preis Oncol, 2017; https://cbioportal.org; **ARID2** K119Lfs*91; **ARID2** Q1194*; **ARID2** Q1313*; **ARID2** Q490*; **ARID2** V681Ffs*25; **ATR** L149*; **CDC73** X473_splice; **CTCF** Q639*; **DICER1** I446Ffs*12; **GNAS** R201H; **GRIN2A** W378*; **IDH1** R132C; **IDH2** R172K; **KDR** E1097K; **KDR** R1032Q; **KMT2C** Q2462*; **KMT2D** Q3892*; **KMT2D** R2734*; **MEN1** X267_splice; **NOTCH4** Q709*; **NOTCH4** W306*; **PBRM1** R710*; **PBRM1** W1076*; **PTPRD** R1088C; **PTPRD** R1496*; **PTPRD** X227_splice; **PTPRT** X950_splice; **PTPRT** Y860*; **RNF43** P77Qfs*6; **SDHB** R90*; **SETD2** Q1619*; **SETD2** Q757*; **SETD2** R1592*; **SF3B1** E902K; **TET2** R1261C; **TP63** R379C

5. Shi, H.; Moriceau, G.; Kong, X.; Lee, M.K.; Lee, H.; Koya, R.C.; Ng, C.; Chodon, T.; Scolyer, R.A.; Dahlman, K.B.; Sosman, J.A.; Kefford, R.F.; Long, G.V.; Nelson, S.F.; Ribas, A.; Lo, R.S. Melanoma whole-exome sequencing identifies (V600E)B-RAF amplification-mediated acquired B-RAF inhibitor resistance. *Nat. Commun*. **2012,** *3*:724. doi: 10.1038/ncomms1727 (**BRAF** gene amplification splice variants)

6. Vido, M.J.; Le, K.; Hartsough, E.J.; Aplin, A.E. BRAF Splice Variant Resistance to RAF Inhibitor Requires Enhanced MEK Association. Cell Rep. **2018**, *25*, 1501-1510. doi: 10.1016/j.celrep.2018.10.049 (**BRAF** gene amplification splice variants)

7. Smalley, K.S.; Lioni, M.; Dalla Palma, M.; Xiao, M.; Desai, B.; Egyhazi, S.; Hansson, J.; Wu, H.; King, A.J.; Van Belle, P.; Elder, D.E.; Flaherty, K.T.; Herlyn, M.; Nathanson, K.L. Increased cyclin D1 expression can mediate BRAF inhibitor resistance in BRAF V600E-mutated melanomas. *Mol. Cancer Ther.* **2008**, *7*, 2876-2883. doi: 10.1158/1535-7163.MCT-08-0431 (**CCND1** gene amplification)

8. Moriceau, G.; Hugo, W.; Hong, A.; Shi, H.; Kong, X.; Yu, C.C.; Koya, R.C.; Samatar, A.A.; Khanlou, N.; Braun, J.; Ruchalski, K.; Seifert, H.; Larkin, J.; Dahlman, K.B.; Johnson, D.B.; Algazi, A.; Sosman, J.A.; Ribas, A.; Lo, R.S. Tunable-combinatorial mechanisms of acquired resistance limit the efficacy of BRAF/MEK cotargeting but result in melanoma drug addiction. *Cancer Cell* **2015**, *27*, 240-256. doi: 10.1016/j.ccell.2014.11.018 (**DUSP4** deletion)

9. Van Allen, E.M.; Wagle, N.; Sucker, A.; Treacy, D.J.; Johannessen, C.M.; Goetz, E.M.; Place, C.S.; Taylor-Weiner, A.; Whittaker, S.; Kryukov, G.V.; Hodis, E.; Rosenberg, M.; McKenna, A.; Cibulskis, K.; Farlow, D.; Zimmer, L.; Hillen, U.; Gutzmer, R.; Goldinger, S.M.; Ugurel, S.; Gogas, H.J.; Egberts, F.; Berking, C.; Trefzer, U.; Loquai, C.; Weide, B.; Hassel, J.C.; Gabriel, S.B.; Carter, S.L.; Getz, G.; Garraway, L.A.; Schadendorf, D.; Dermatologic Cooperative Oncology Group of Germany (DeCOG). The genetic landscape of clinical resistance to RAF inhibition in metastatic melanoma. *Cancer Discov.* **2014**, *4*, 94-109. doi: 10.1158/2159-8290.CD-13-0617 (**HOXD8** nonsense variant; **MAP2K1** C121S; **MAP2K1** C387A; **MAP2K1** F129L; **MAP2K1** F53L; **MAP2K1** G128V; **MAP2K1** Q56P; **MAP2K1** V60E; **MAP2K2** C125S; **MAP2K2** L46F; **MAP2K2** N126D; **MAP2K2** Q60P; **MAP2K2** V35M; **MITF** gene amplification; **RAC1** P29S)

10. Dietrich, P.; Kuphal, S.; Spruss, T.; Hellerbrand, C.; Bosserhoff, A.K. Wild-type KRAS is a novel therapeutic target for melanoma contributing to primary and acquired resistance to BRAF inhibition. *Oncogene.* **2018**, *37*, 897-911. doi: 10.1038/onc.2017.391 (**KRAS** Q61H; **KRAS** G12C/R)

11. Wagle, N.; Emery, C.; Berger, M.F.; Davis, M.J.; Sawyer, A.; Pochanard, P.; Kehoe, S.M.; Johannessen, C.M.; Macconaill, L.E.; Hahn, W.C.; Meyerson, M.; Garraway, L.A. Dissecting therapeutic resistance to RAF inhibition in melanoma by tumor genomic profiling. *J Clin Oncol.* **2011**, *29*, 3085-3096. doi: 10.1200/JCO.2010.33.2312 (**MAP2K1** C121S; **MAP2K1** C387A; **MAP2K1** F129L; **MAP2K1** F53L; **MAP2K1** G128V; **MAP2K1** Q56P; **MAP2K1** V60E)

12. Trunzer, K.; Pavlick, A.C.; Schuchter, L.; Gonzalez, R.; McArthur, G.A.; Hutson, T.E.; Moschos, S.J.; Flaherty, K.T.; Kim, K.B.; Weber, J.S.; Hersey, P.; Long, G.V.; Lawrence, D.; Ott, P.A.; Amaravadi, R.K.; Lewis, K.D.; Puzanov, I.; Lo, R.S.; Koehler, A.; Kockx, M.; Spleiss, O.; Schell-Steven, A.; Gilbert, H.N.; Cockey, L.; Bollag, G.; Lee, R.J.; Joe, A.K.; Sosman, J.A.; Ribas, A. Pharmacodynamic effects and mechanisms of resistance to vemurafenib in patients with metastatic melanoma. *J. Clin. Oncol*. **2013**, *31*, 1767-1774. doi: 10.1200/JCO.2012.44.7888 (**MAP2K1** C121S; **MAP2K1** C387A; **MAP2K1** F129L; **MAP2K1** F53L; **MAP2K1** G128V; **MAP2K1** Q56P; **MAP2K1** V60E; **NRAS** G12D/R; **NRAS** G13R; **NRAS** Q61K/R/L)

13. Ahronian, L.G.; Sennott, E.M.; Van Allen, E.M.; Wagle, N.; Kwak, E.L.; Faris, J.E.; Godfrey, J.T.; Nishimura, K.; Lynch, K.D.; Mermel, C.H.; Lockerman, E.L.; Kalsy, A.; Gurski, J.M. Jr, Bahl, S.; Anderka, K.; Green, L.M.; Lennon, N.J.; Huynh, T.G.; Mino-Kenudson, M.; Getz, G.; Dias-Santagata, D.; Iafrate, A.J.; Engelman, J.A.; Garraway, L.A.; Corcoran, R.B. Clinical Acquired Resistance to RAF Inhibitor Combinations in BRAF-Mutant Colorectal Cancer through MAPK Pathway Alterations. *Cancer Discov.* **2015**, *5*, 358-367. doi: 10.1158/2159-8290.CD-14-1518 **(MAP2K1** C121S; **MAP2K1** C387A; **MAP2K1** F129L; **MAP2K1** F53L; **MAP2K1** G128V; **MAP2K1** Q56P; **MAP2K1** V60E)

14. Villanueva, J.; Infante, J.R.; Krepler, C.; Reyes-Uribe, P.; Samanta, M.; Chen, H.Y.; Li, B.; Swoboda, R.K.; Wilson, M.; Vultur, A.; Fukunaba-Kalabis, M.; Wubbenhorst, B.; Chen, T.Y.; Liu, Q.; Sproesser, K.; DeMarini, D.J.; Gilmer, T.M.; Martin, A.M.; Marmorstein, R.; Schultz, D.C.; Speicher, D.W.; Karakousis, G.C.; Xu, W.; Amaravadi, R.K.; Xu, X.; Schuchter, L.M.; Herlyn, M.; Nathanson, K.L. Concurrent MEK2 mutation and BRAF amplification confer resistance to BRAF and MEK inhibitors in melanoma. *Cell Rep.* **2013,** *4*, 1090-1099. doi: 10.1016/j.celrep.2013.08.023 **(MAP2K2** C125S; **MAP2K2** L46F; **MAP2K2** N126D; **MAP2K2** Q60P; **MAP2K2** V35M)

15. Whittaker, S.R.; Theurillat, J.P.; Van Allen, E.; Wagle, N.; Hsiao, J.; Cowley, G.S.; Schadendorf, D.; Root, D.E.; Garraway, L.A. A genome-scale RNA interference screen implicates NF1 loss in resistance to RAF inhibition. *Cancer Discov.* **2013**, *3*, 350-362. doi: 10.1158/2159-8290.CD-12-0470 (**NF1** deletion)

16. Krauthammer, M.; Kong, Y.; Ha, B.H.; Evans, P.; Bacchiocchi, A.; McCusker, J.P.; Cheng, E.; Davis, M.J.; Goh, G.; Choi, M.; Ariyan, S.; Narayan, D.; Dutton-Regester, K.; Capatana, A.; Holman, E.C.; Bosenberg, M.; Sznol, M.; Kluger, H.M.; Brash, D.E.; Stern, D.F.; Materin, M.A.; Lo, R.S.; Mane, S.; Ma, S.; Kidd, K.K.; Hayward, N.K.; Lifton, R.P.; Schlessinger, J.; Boggon, T.J.; Halaban, R. Exome sequencing identifies recurrent somatic RAC1 mutations in melanoma. *Nat. Genet.* **2012,** *44*, 1006-1014. doi: 10.1038/ng.2359 (**RAC1** P29S)

17. Antonello, Z.A.; Hsu, N.; Bhasin, M.; Roti, G.; Joshi, M.; Van Hummelen, P.; Ye, E.; Lo, A.S.; Karumanchi, S.A.; Bryke, C.R.; Nucera, C. Vemurafenib-resistance via de novo RBM genes mutations and chromosome 5 aberrations is overcome by combined therapy with palbociclib in thyroid carcinoma with BRAFV600E. *Oncotarget*. **2017**, *8*, 84743-84760. doi: 10.18632/oncotarget.21262 (**RBM10** E513del; **RBMX** P167A)

Table S2. Average library size and final coverage of whole-exome sequencing of genomic DNA samples extracted from vemurafenib- (PLXR) or trametinib-resistant (TRAR) cell lines.

Cell population	Library size (average)	Average throughput depth of target regions
11_PLXR	329 bp	109x
11_TRAR	335 bp	106x
21_PLXR	359 bp	137x
21_TRAR	354 bp	142x
28_PLXR	350 bp	173x
28_TRAR	346 bp	115x
29_PLXR	340 bp	126x
29_TRAR	334 bp	120x
12_PLXR	338 bp	122x
17_TRAR	337 bp	102x

The original immunoblots. Cropped bands are shown in frames. Protein levels were normalized to GAPDH or β-actin based on densitometry analysis, and relative protein levels are shown.

Corresponding to Figure 1E

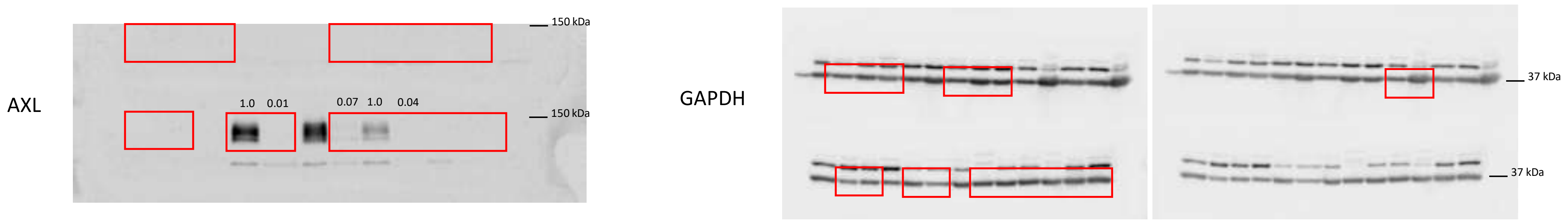

Corresponding to Figure 2C

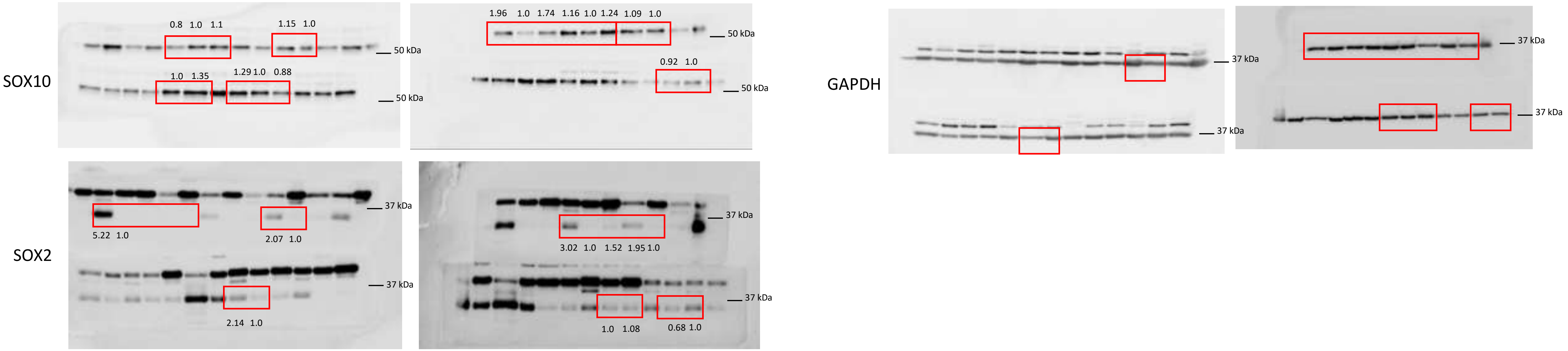

Corresponding to Figure 3B

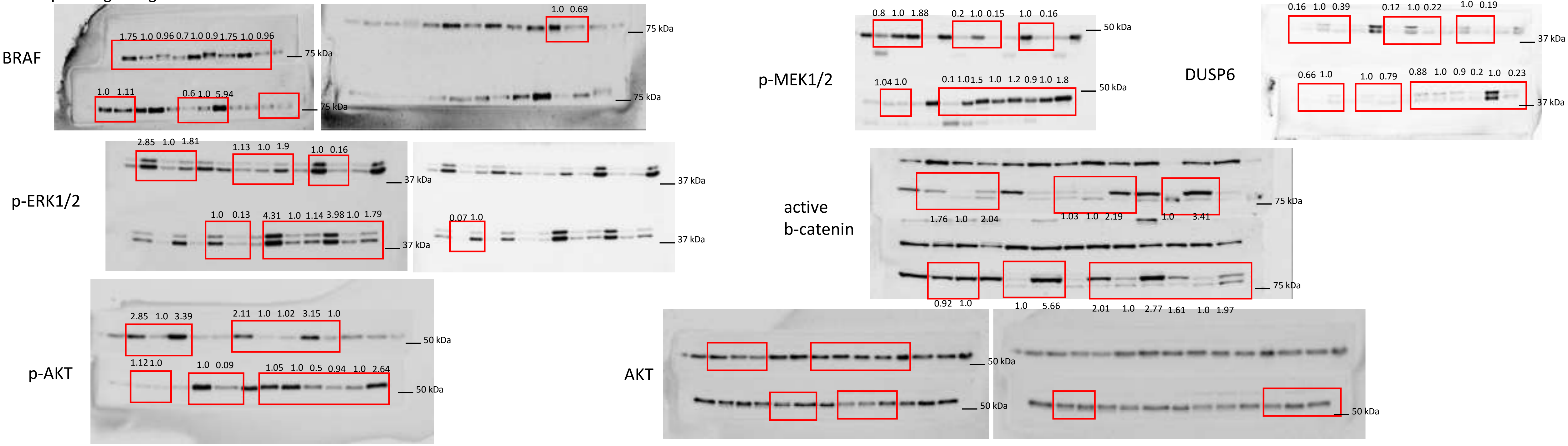

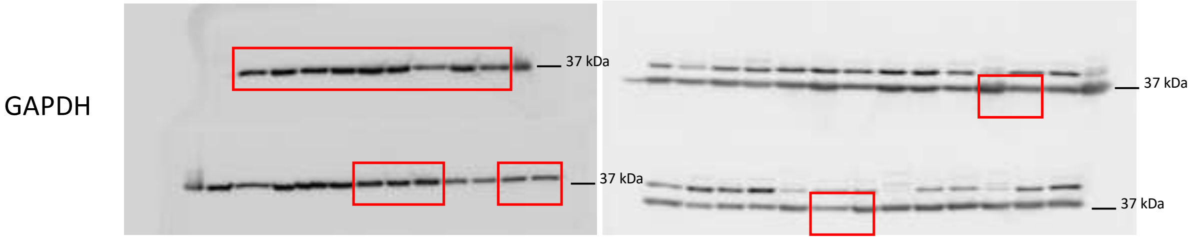

Corresponding to Figure 4B

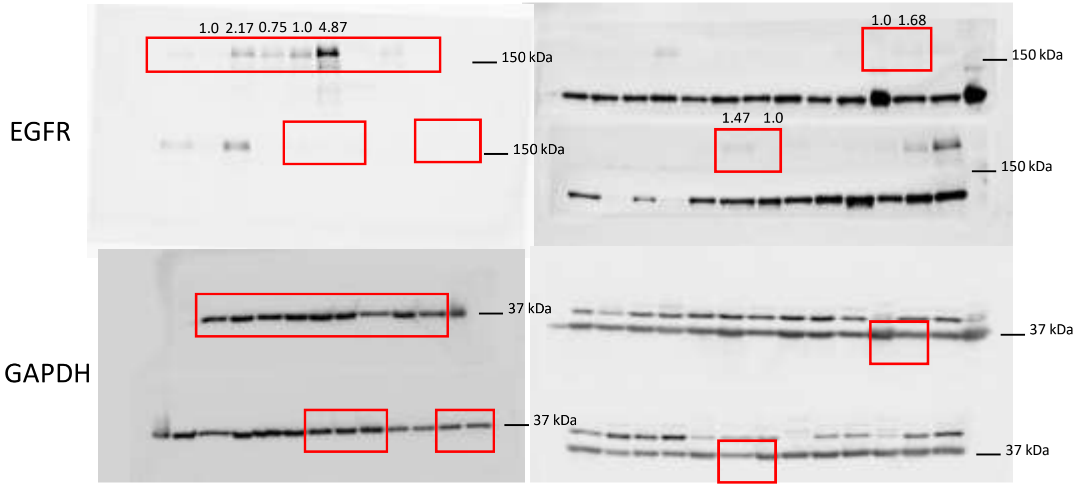

Corresponding to Figure 4D

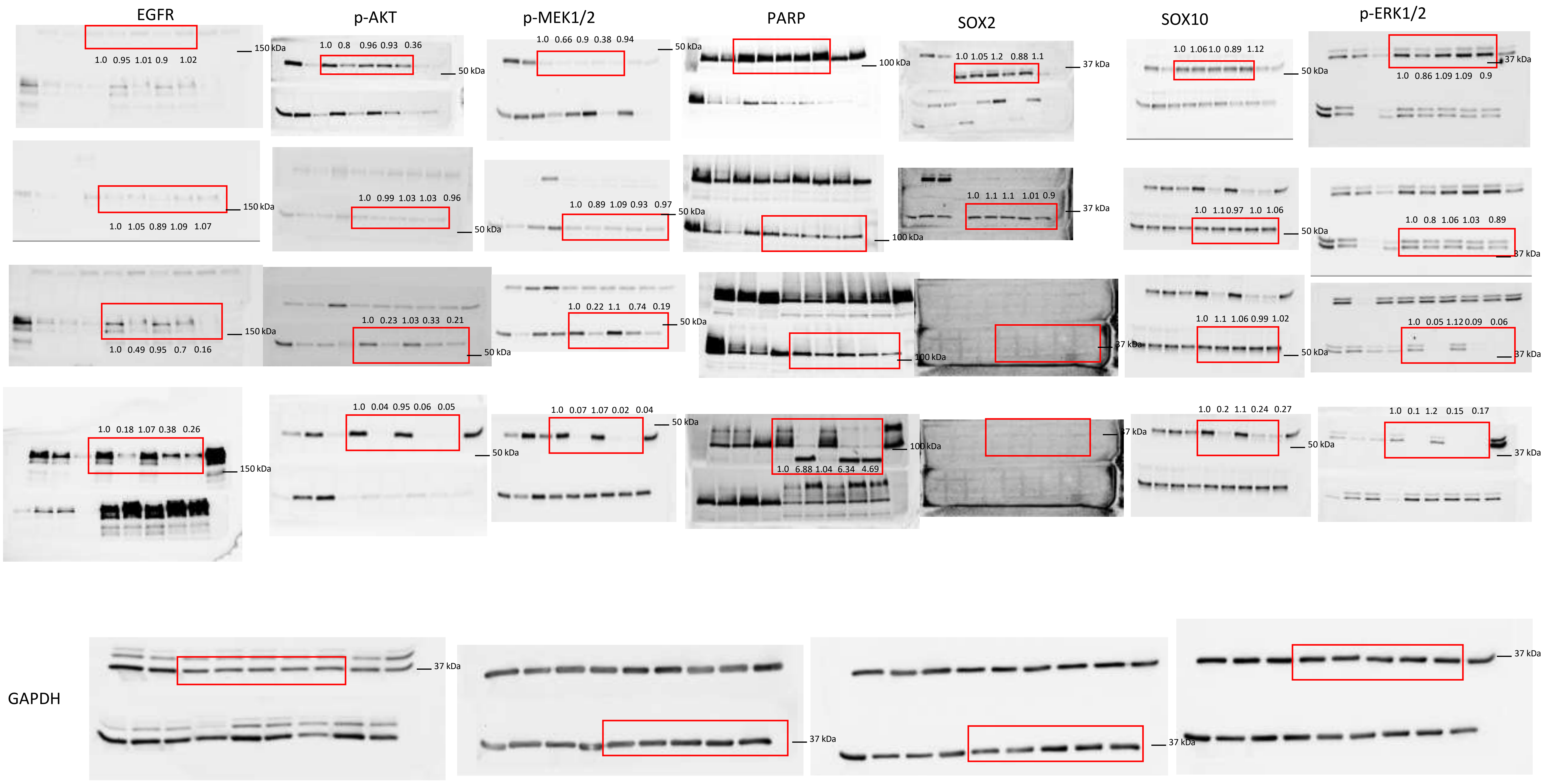

Corresponding to Figure 5A

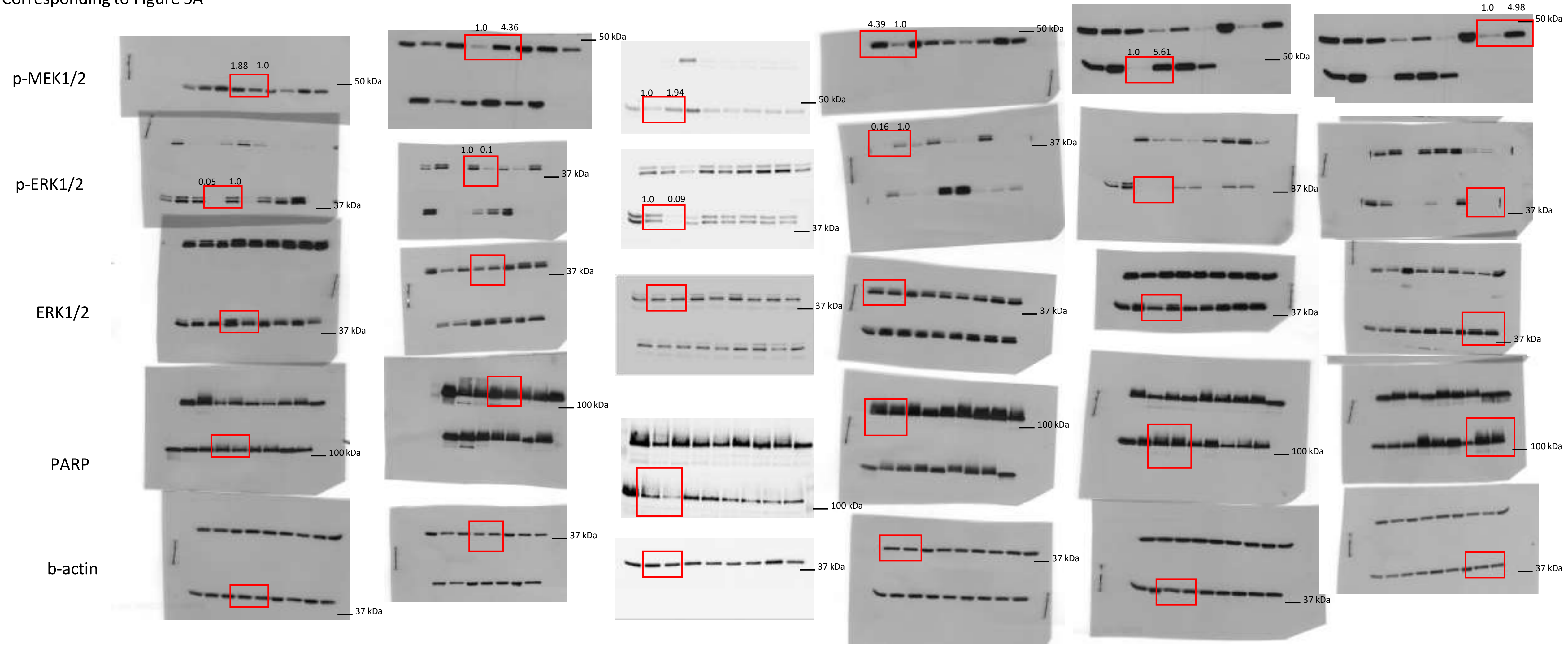

Corresponding to Figure 5A (continued)

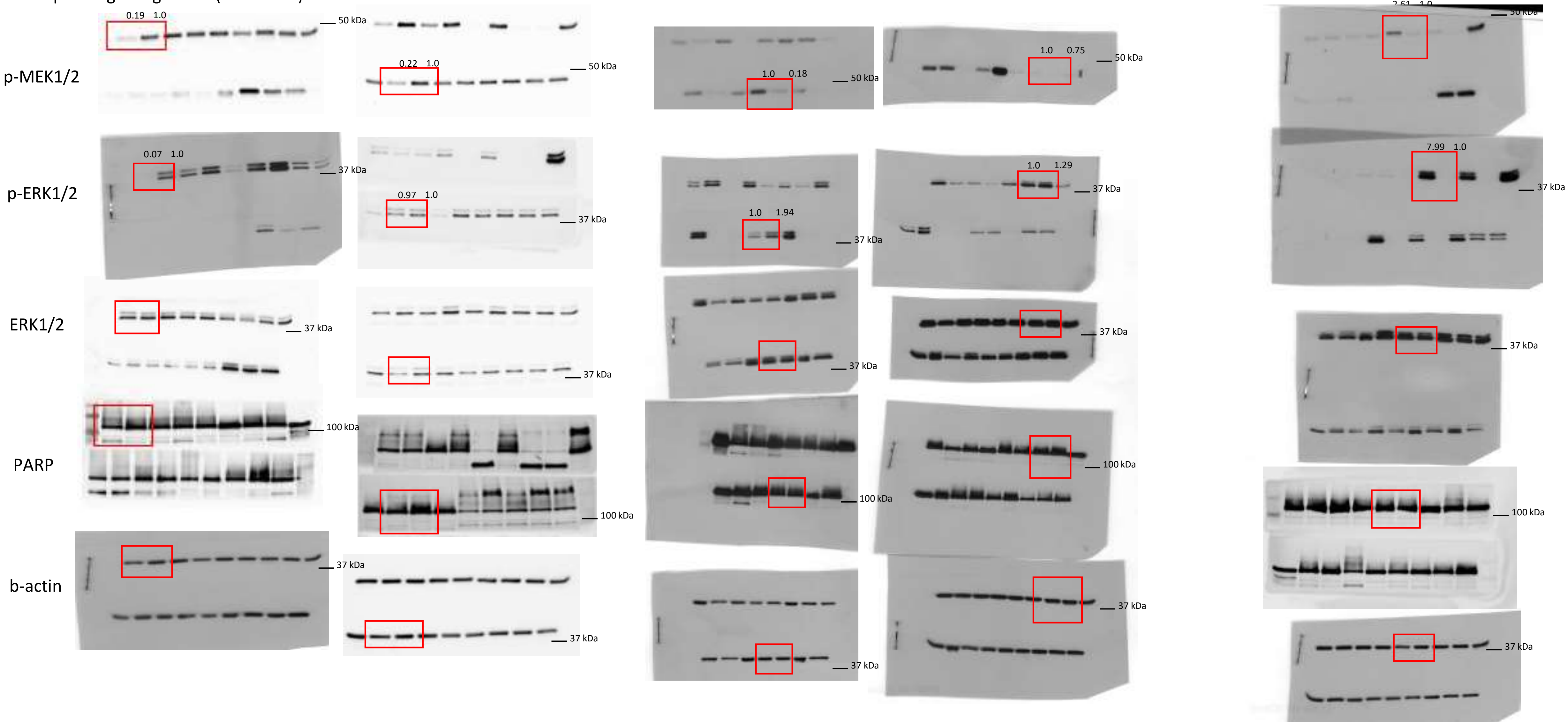

Corresponding to Figure 6B

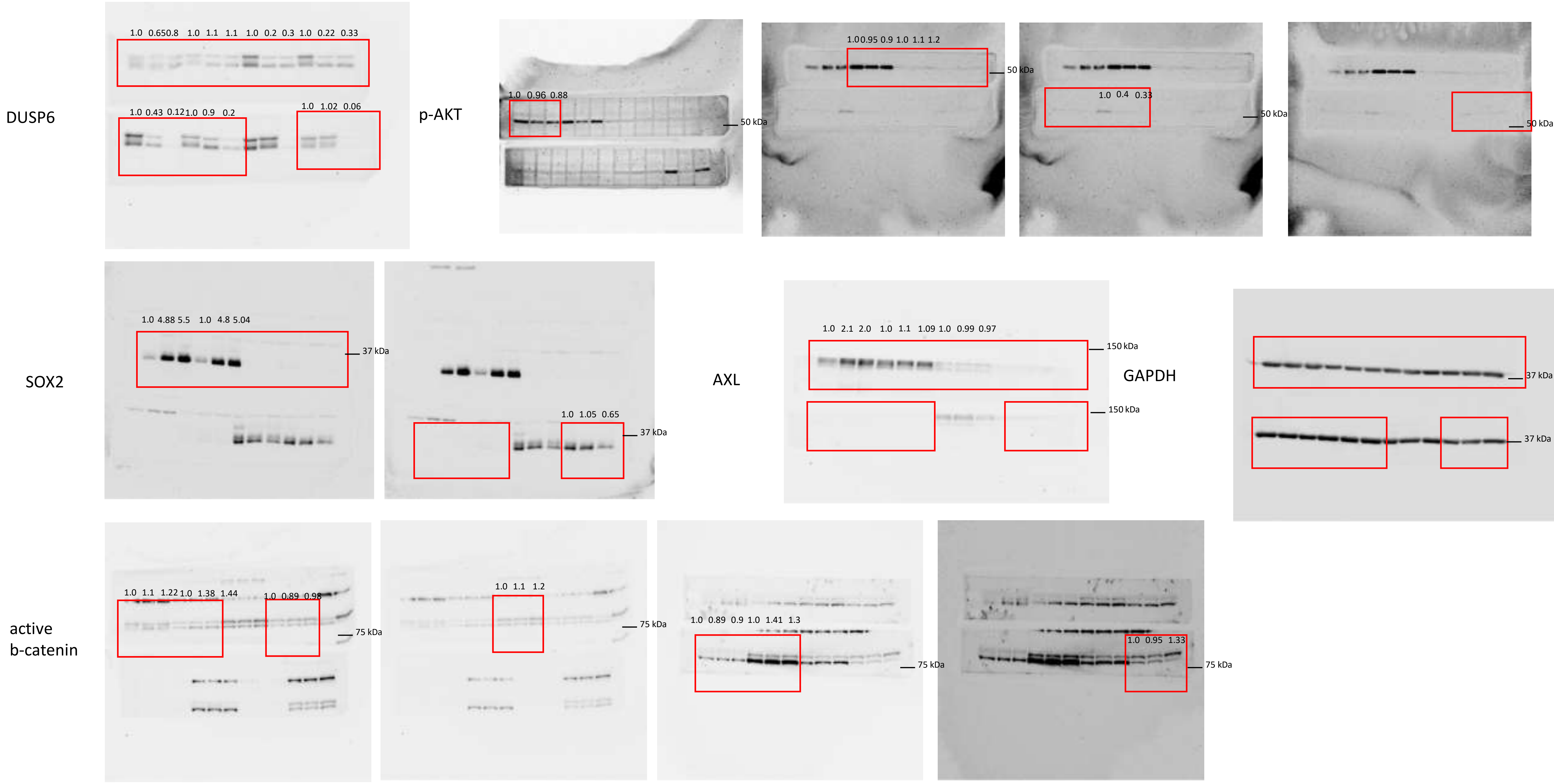

The original Human Phospho-Kinase Array membranes corresponding to Figures 3A and 6A.

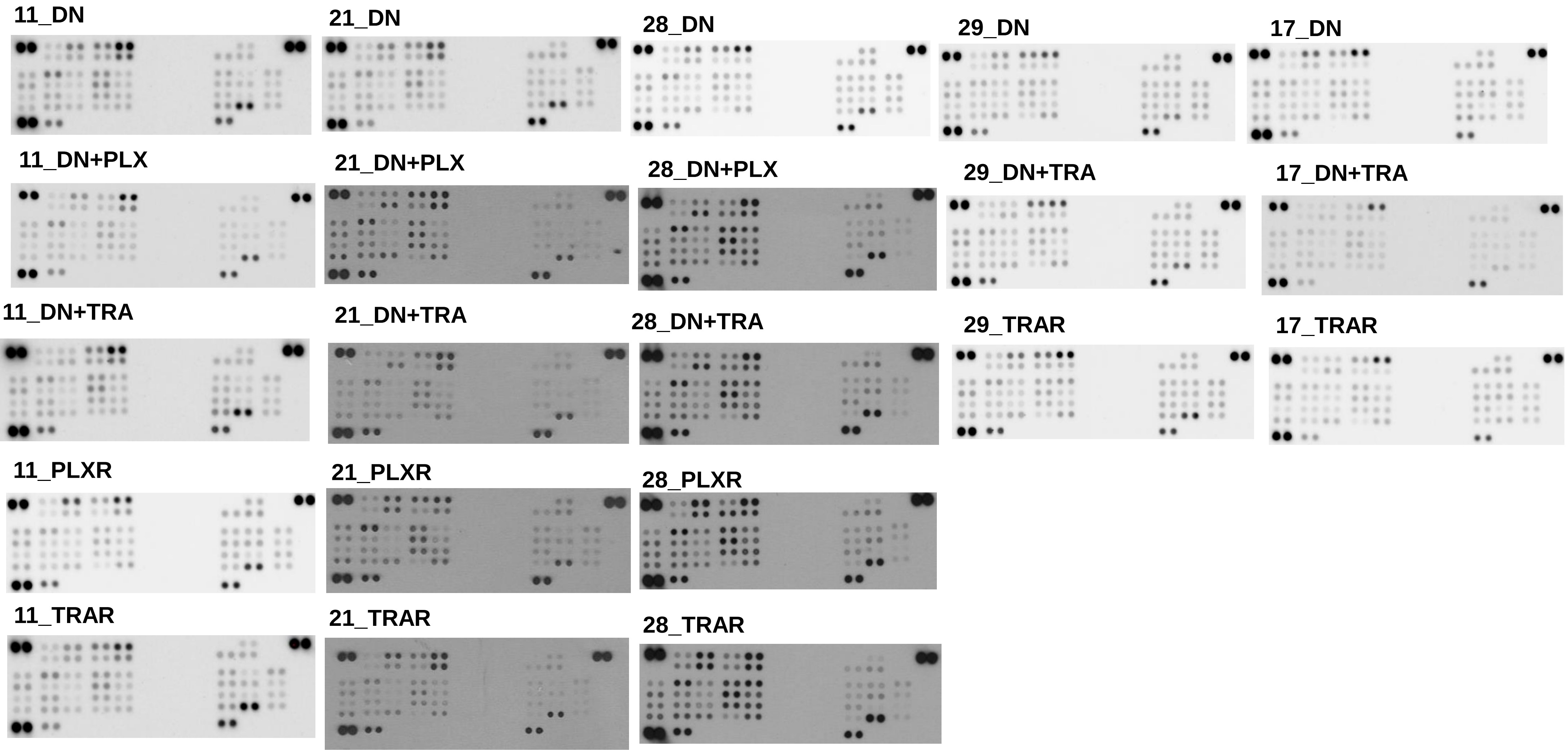

Supplement: Supplementary file 1 [file cells-09-00142-s001.pdf]
